# Correction: Coronavirus Cell Entry Occurs through the Endo-/Lysosomal Pathway in a Proteolysis-Dependent Manner

**DOI:** 10.1371/journal.ppat.1004709

**Published:** 2015-02-13

**Authors:** The PLOS Pathogens Staff

## Notice of Republication

This article was republished on December 15, 2014 to correct an error with Figure 6. The prior version contained a copy of Figure 8 in place of Figure 6. Please download this article again to view the correct version. The originally published, uncorrected article and the republished, corrected article are provided here for reference.

## Supporting Information

S1 File Originally published, uncorrected article. (PDF)

S2 File Republished, corrected article. (PDF)
