# Supplementary material for: Coronavirus Cell Entry Occurs through the Endo-/Lysosomal Pathway in a Proteolysis-Dependent Manner
Source: PLoS Pathog. 2014 Nov 6;10(11):e1004502. doi: 10.1371/journal.ppat.1004502 (PMC4223067; doi:10.1371/journal.ppat.1004502)
Supplement: Text S1 — Supporting information. (PDF) [file ppat.1004502.s001.pdf]

Supporting Information

Coronavirus cell entry occurs through the endo-/lysosomal pathway in a proteolysis-dependent manner

Christine Burkard[1,✦], Monique H Verheije[1,#], Oliver Wicht[1], Sander I van Kasteren[2], Frank J van Kuppeveld[1], Bart L Haagmans[3], Lucas Pelkmans[4], Peter J M Rottier[1], Berend Jan Bosch[1], Cornelis A M de Haan[1,*]

[1]Virology Division, Department of Infectious Diseases and Immunology, Faculty of Veterinary Medicine, Utrecht University, Utrecht, The Netherlands
[2]Division of bio-organic synthesis, Leiden Institute of Chemistry, Leiden University, Leiden, The Netherlands
[3] Department of Viroscience, Erasmus MC, Rotterdam, The Netherlands
[4]Institute of Molecular Life Sciences, University of Zurich, Zurich, Switzerland
* Corresponding author

✦ Current address: The Roslin Institute and Royal (Dick) School of Veterinary Studies, University of Edinburgh, Easter Bush, Edinburgh EH25 9RG, UK
# Current address: Department of Pathobiology, Division Pathology, Faculty of Veterinary Medicine, Utrecht University, Utrecht, The Netherlands

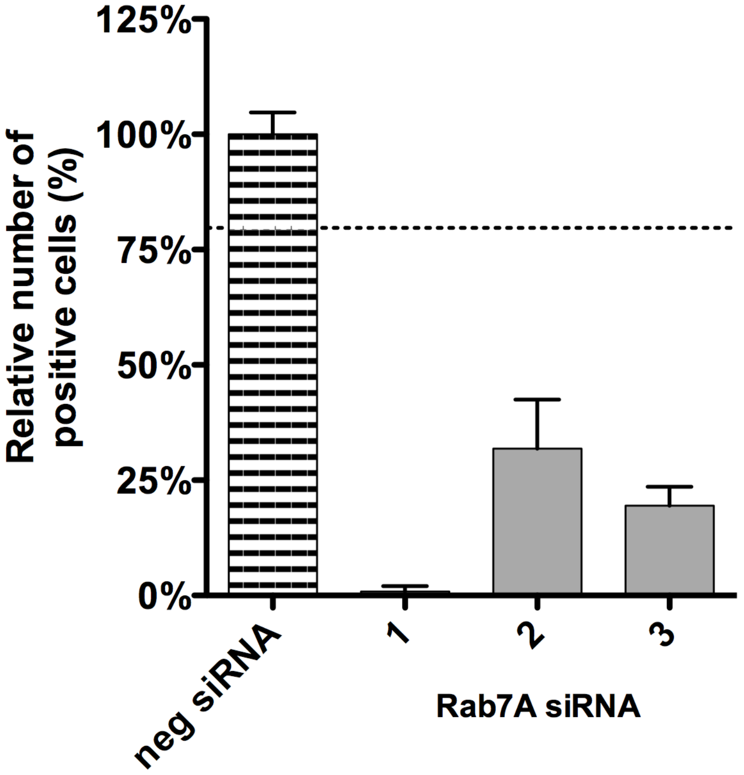

Figure S1. Confirmation of protein knockdown upon Rab7A siRNA transfection. Cells were simultaneously transfected with mRFP-Rab7A plasmid and siRNAs against Rab7A. 24h post transfection cells were fixed and the number of positive cells assessed. Dotted line shows the lower 95% confidence interval of the negative siRNA control. Error bars represent SEM, n=3.

.

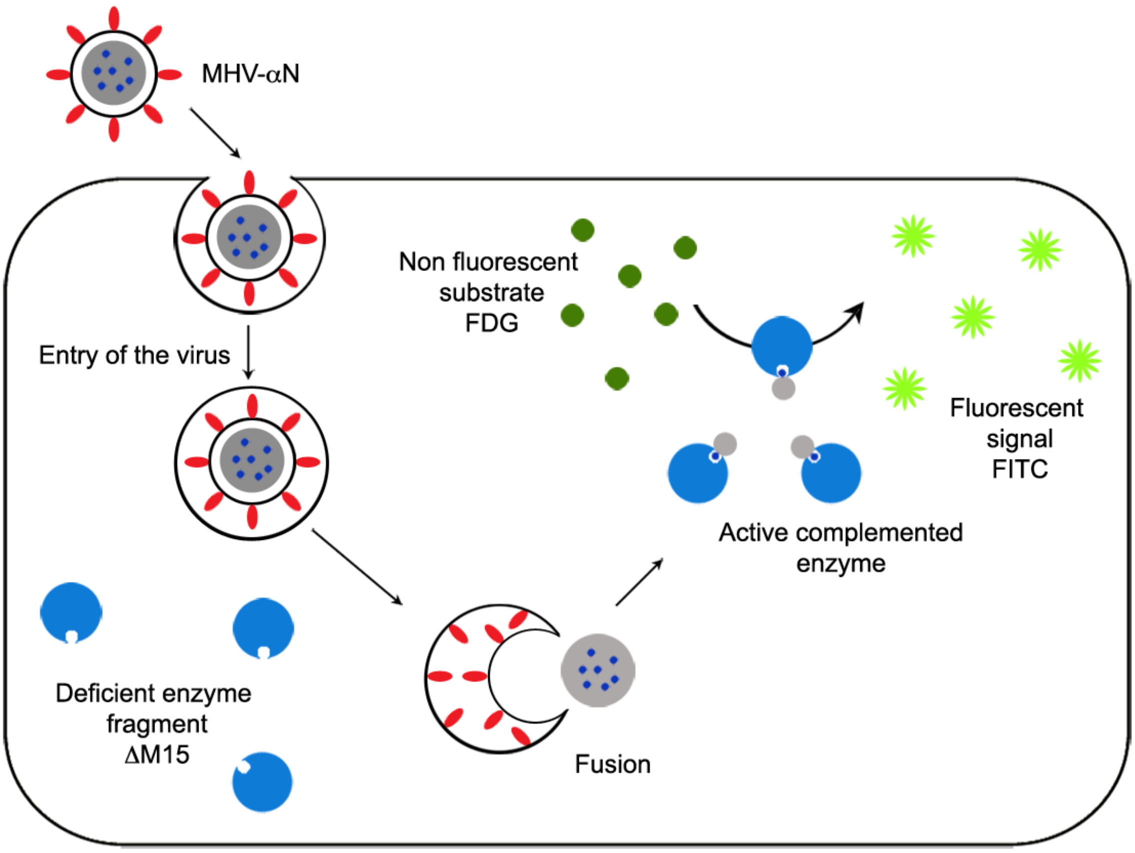

Figure S2. Replication-independent fusion assay. Recombinant MHV containing a nucleocapsid protein with a 45-aa α-peptide extension (αN) are bound and internalized into the target cells. Upon fusion the nucleocapsid proteins are released into the cytosol where the deficient, inactive β-galactosidase enzyme ΔM15 is present. The ΔM15 is subsequently complemented by the α-peptide exposed by the N protein, thereby reconstituting an active enzyme. This enzyme can now convert the FDG substrate fluorescein, the production of which can be measured by FACS or fluorescence microscopy.

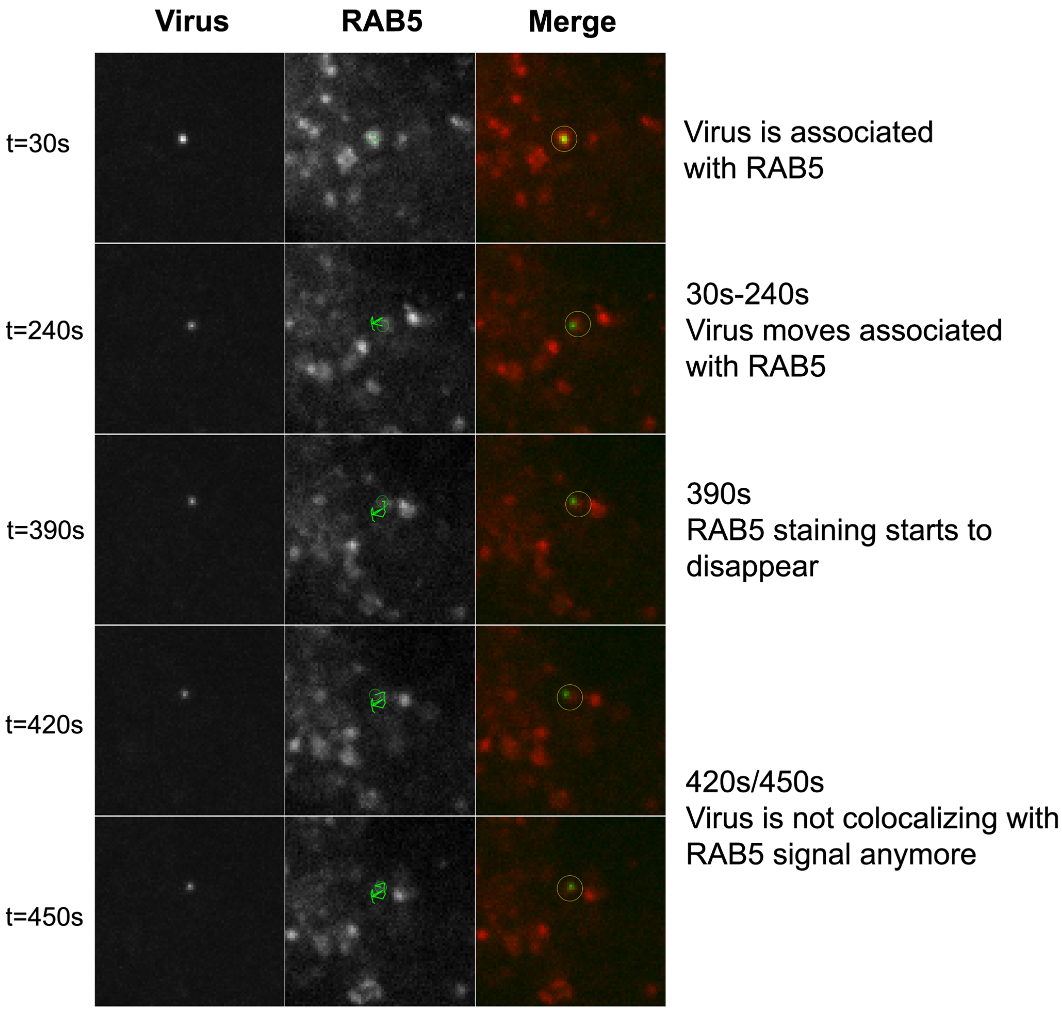

Figure S3. Stills from live-cell co-localization studies of MHV-DL488 with RAB5. Green fluorescently-labeled MHV-DL488 virus was bound to RAB5-mRFP expressing HeLa-mCC1a cells at 4°C at MOI=20 for 90min. Inoculation medium was replaced by warm, trypan blue-containing medium, which shifts the emission spectrum of surface bound particles and thereby renders them undetectable in the 505-530nm channel [64]. Cells were imaged using a spinning-disc confocal microscope acquiring z-stacks in 30s intervals over 10min time intervals from 10-70min post warming. Virus particles were automatically detected and circled in the green channel. Upon overlay of the selected virion areas with the red channel co-localization was assessed by measurement of the underlying pixel density. Virion and endosomal vesicle movement were manually tracked separately in x/y- and z-direction. Co-localization over time was analyzed and scored (Fig. 4). A virion is shown, which is initially co-localizing/associating with RAB5. The virus moves together with the vesicle in x/y- and z-direction. 390s after the start of the recording the RAB5 staining surrounding the virion starts to disappear, indicating that the virus is now dissociated from the RAB5-positive vesicle (classified as 'Assoc/Dissoc' in Fig. 4B).

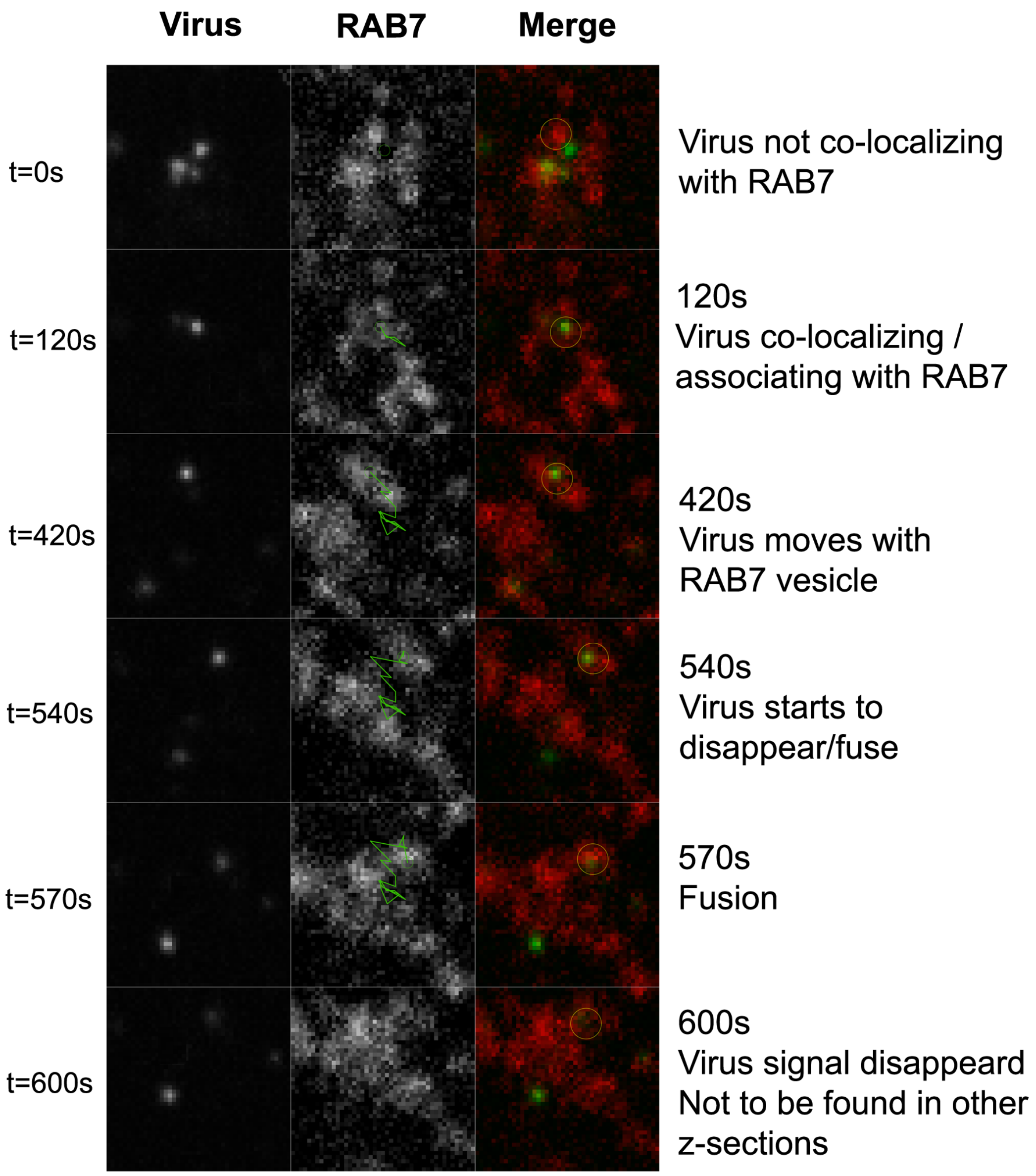

Figure S4. Stills from live-cell co-localization studies of MHV-DL488 with RAB7. Live cell imaging was performed as described in the legend to supplementary Fig. S2, using cells expressing RAB7-mRFP instead of RAB5-mRFP. A virion is shown, which is initially not co-localizing with a RAB7 vesicle. 120s after the start of the recording the virus associates with the RAB7-positive late endosomal/lysosomal (LE/LY) vesicle. The virus moves together with the vesicle in x/y- and z direction until about t=540s, after which the green fluorescence of the virus starts to disappear. At t=600 sec, the green fluorescence has disappeared completely indicating that the virus has fused with the late endosomal/lysosomal compartment (classified as 'Fusing' in Fig. 4B).

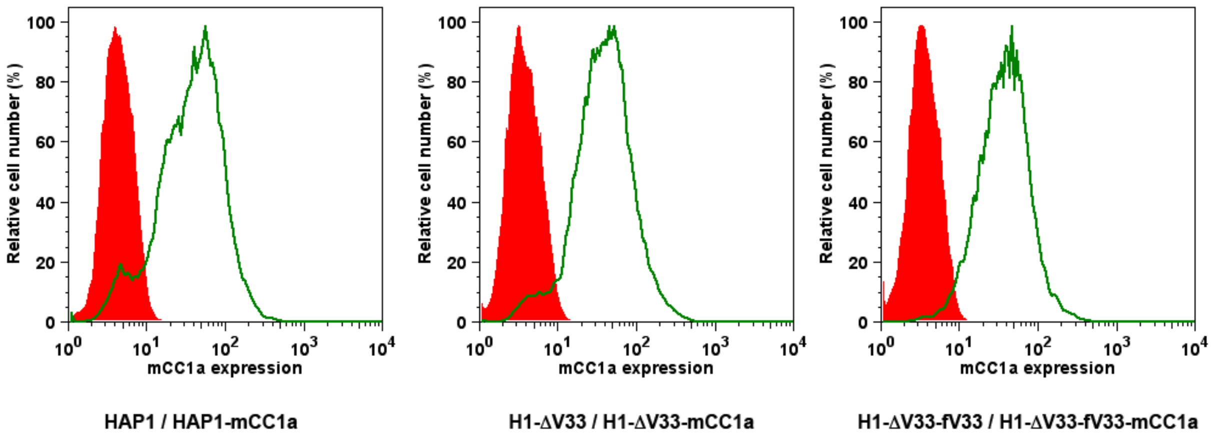

Figure S5. Confirmation of even expression of mCeacam in haploid cells. HAP1, H1-ΔV33, and H1-ΔV33-fV33 cells and their stably mCeacam1a expressing counterparts were immunostained using N-CEACAM-Fc [80] primary and secondary AF488 goat anti-rabbit antibody and analyzed by FACS. Left panel shows HAP1 (red) and HAP1-mCC1a cells (green), middle H1-ΔV33 (red) and H1-ΔV33-mCC1a (green), right H1-ΔV33-fV33 (red) and H1-ΔV33-fV33-mCC1a (green).

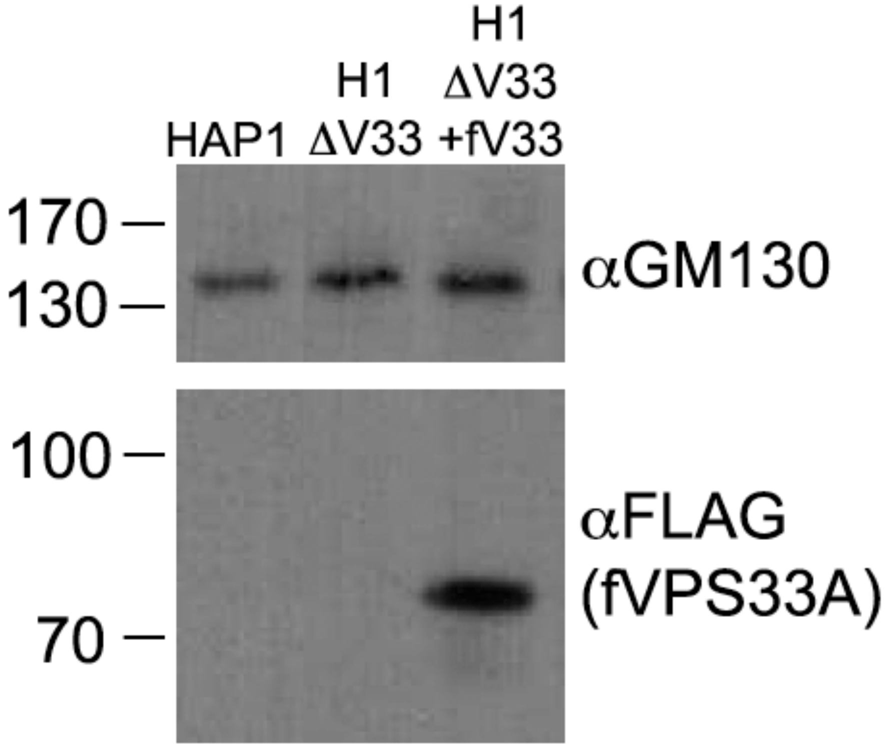

Figure S6. Confirmation of re-transfection of FLAG-VPS33A. Lysates of HAP1, H1-ΔV33, and H1-ΔV33-fV33 cells, the latter stably re-transfected with FlAG-VPS33A, were subjected to immunoblotting after gel electrophoresis. Antibodies used were against FLAG and GM130, the latter to control the loading, were used.

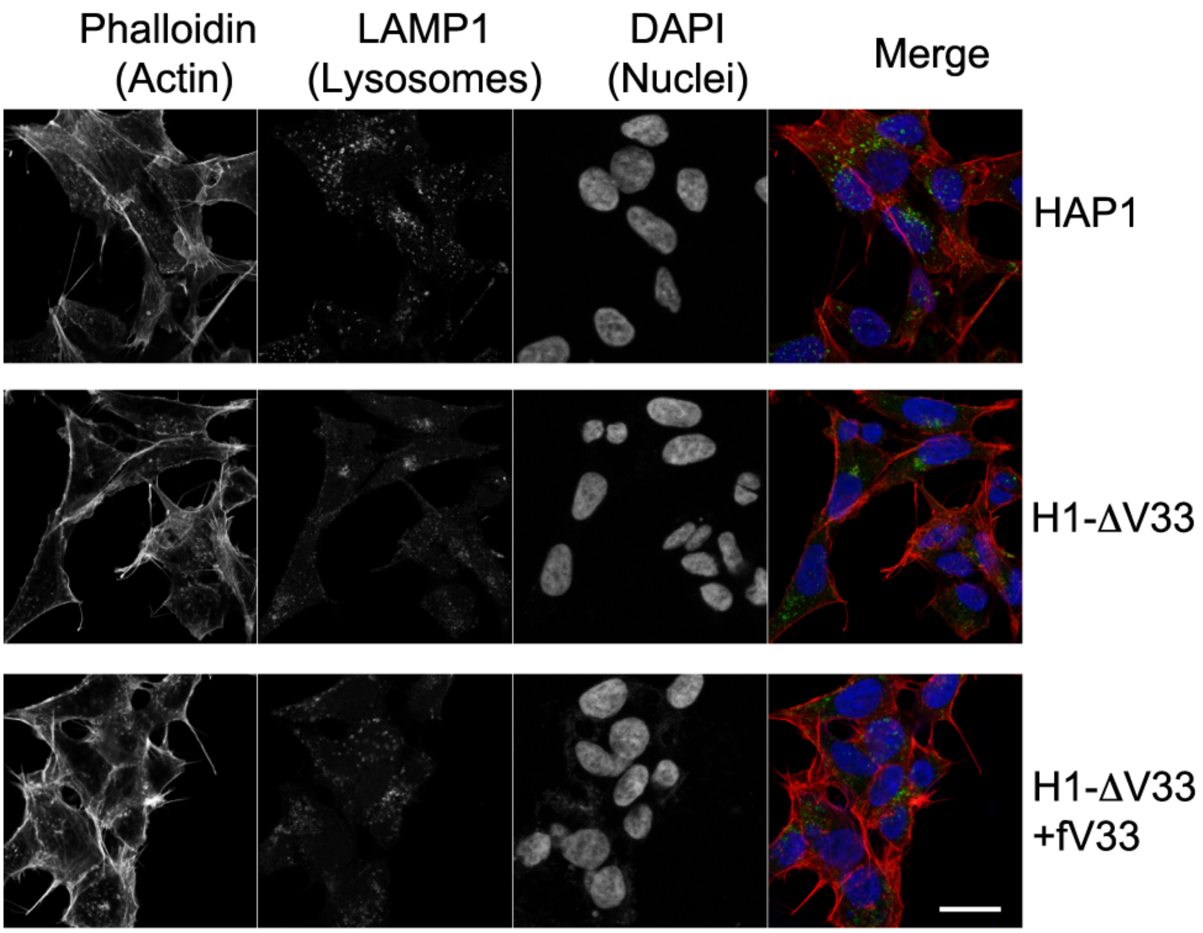

Figure S7: Localization of lysosomes is affected in haploid cells lacking VPS33A. HAP1, H1-ΔV33, and H1-ΔV33-fV33 cells were fixed and stained with rabbit anti-LAMP1 and AF488-conjugated anti-rabbit, AF568-conjugated Phalloidin, and DAPI. Cells were analyzed by confocal microscopy. Scale bar indicates 20μm.

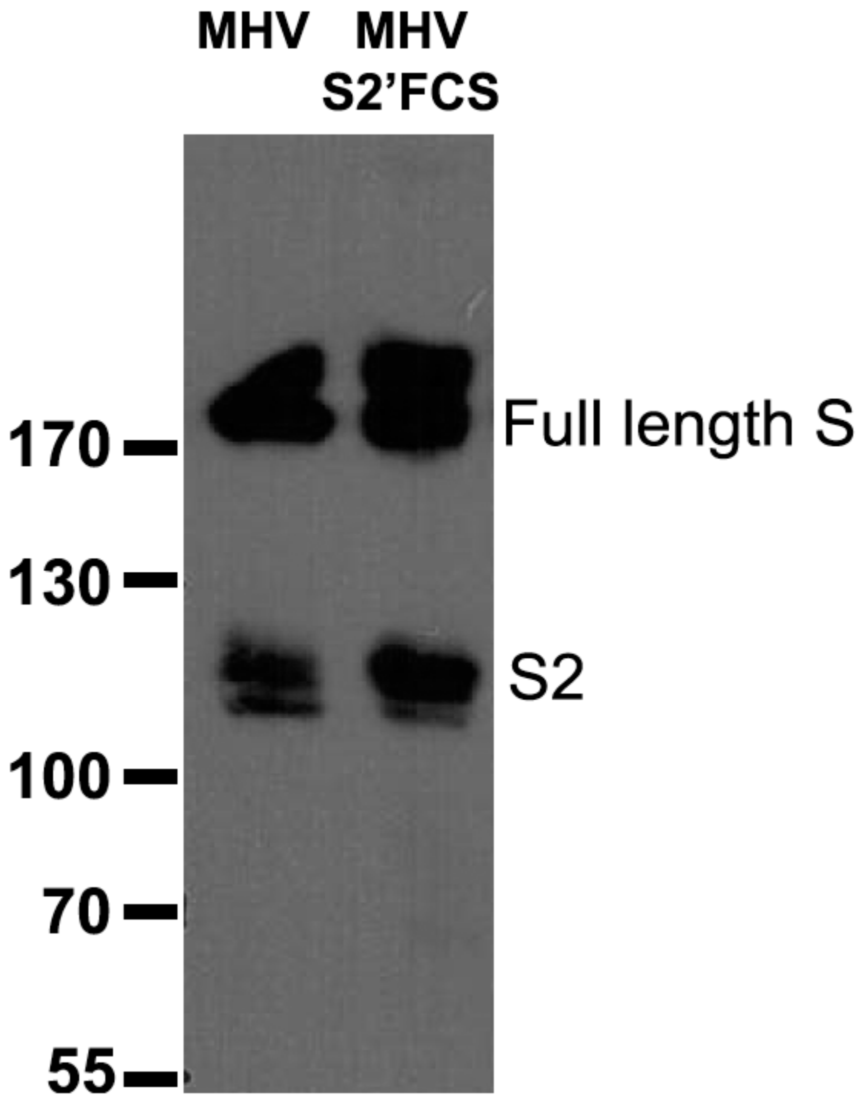

Figure S8. Western blot analysis of spike proteins of purified MHV and MHV-S2’FCS. 20% sucrose cushion purified MHV (MHV) and MHV-S2’FCS were subjected to gel electrophoresis and immunoblotting using antibodies recognizing the carboxy-terminal part of the spike protein. Regardless of the virus preparations used, either the full length S protein or the full length S2 subunit is detected with this antibody. There is no indication that the S protein carrying the FCS is cleaved during biogenesis of the virus ore thereafter.

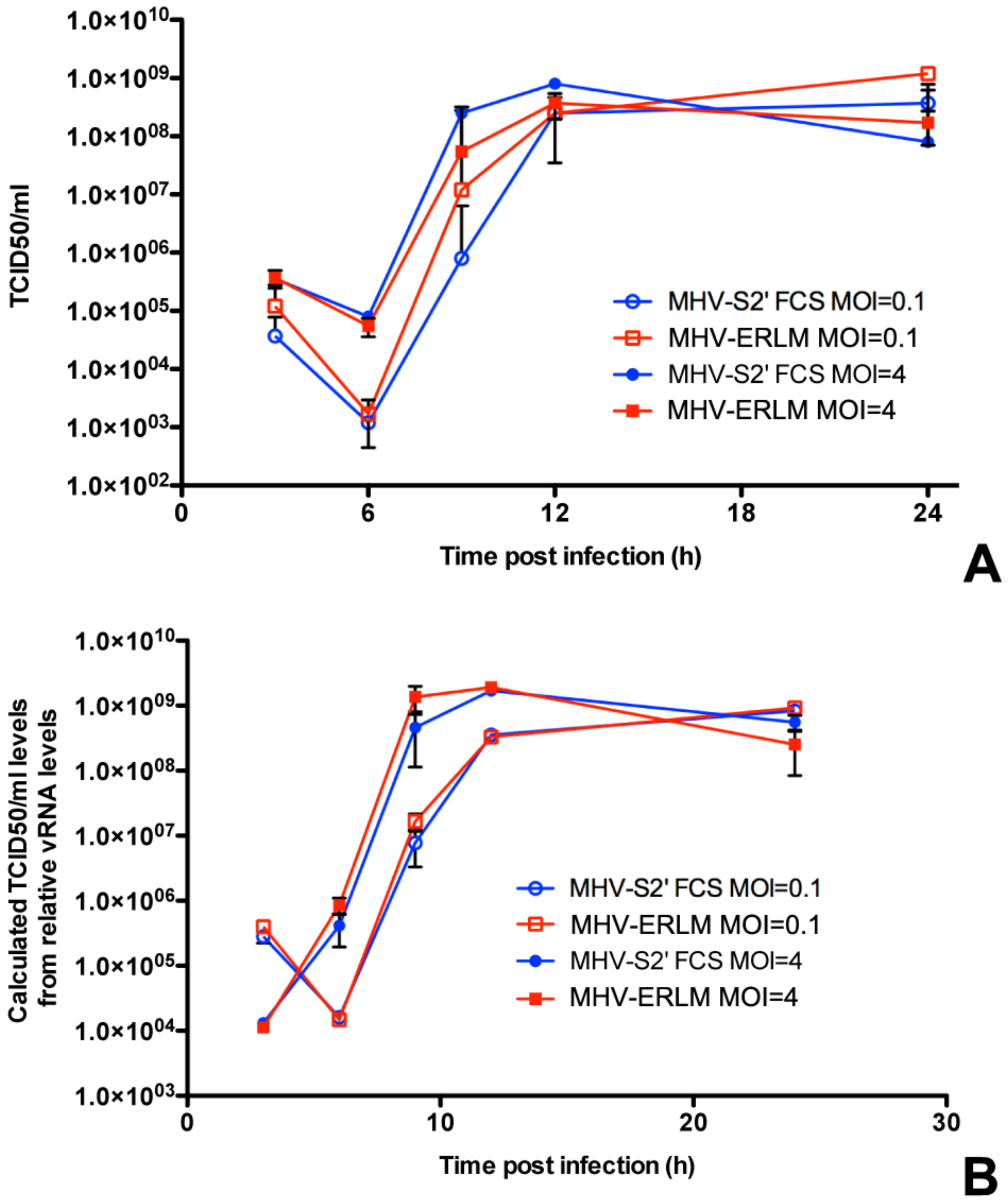

Figure S9. Growth curve of MHV and MHV-S2'FCS. LR7 cells were inoculated with the wild type S containing MHV (MHV-ERLM) or MHV-S2'FCS At the indicated times thereafter, cell culture supernatants were collected for **A)** TCID50 analysis or **B)** measurement of the amount of released viral RNA by qRT-PCR. Error bars represent SEM, n=3

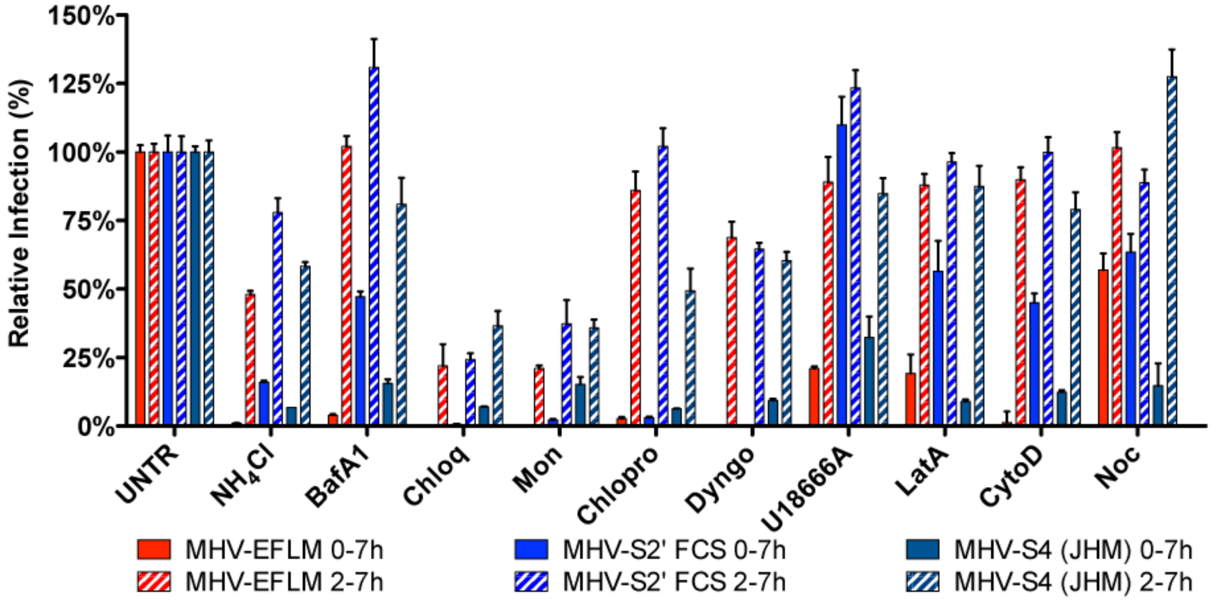

Figures S10. Effects of endocytosis-affecting agents on MHV-S2'FCS and MHV-S4 (JHM) infection. HeLa-mCC1a cells were inoculated with MHV-EFLM, MHV-S2'FCS, or MHV-S4 (JHM) at MOI=0.2 or MOI=0.1 (MHV-S4) for 2h. Cells were (pre-)treated with ammonium chloride (NH4Cl), Bafilomycin A1 (BafA1), Chloroquine (Chloq), Monensin (Mon), Chlorpromazine (Chlopro), Dyngo-4A, U18666A, Latrunculin A, (LatA), Cytochalasin D (DytoD), and Nocodazole (Noc), from 30 min prior to 7h post infection (0-7) or from 2 to 7h post infection (2-7). Infection levels were determined by measuring the luciferase activity in cell lysates relative to mock-treated cells (UNTR). Error bars represent 1 SEM, n=3*3.

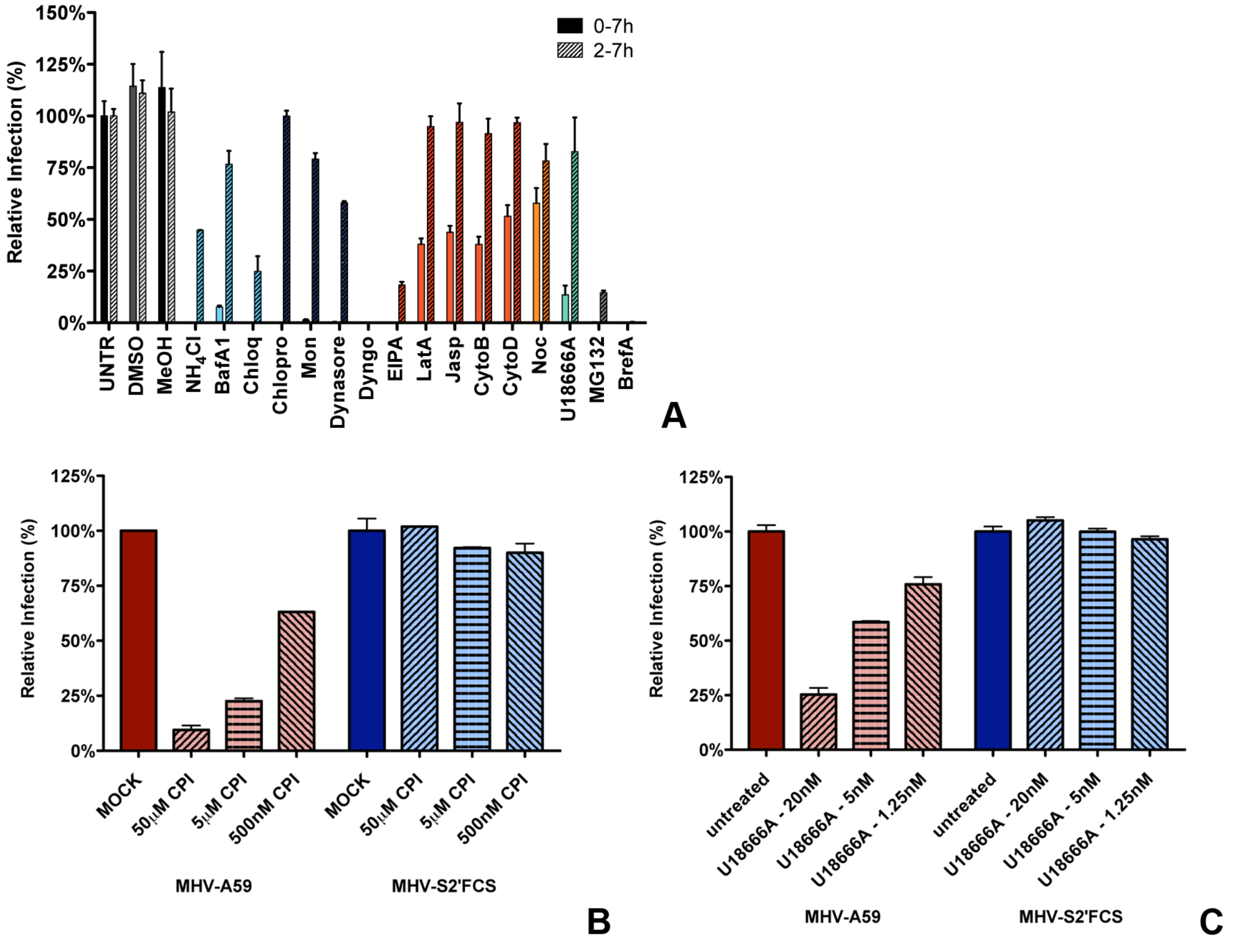

Figure S11. Entry of MHV-A59 in murine LR7 cells also requires endosomal maturation. A) Clathrin-mediated endocytosis and late endosome-to-lysosome trafficking is required for entry of MHV in murine LR7 cells. LR7 cells, inoculated with MHV-EFLM at MOI=0.2, were treated with the different inhibitors from 30 min prior to 7h post inoculation (0-7h) or from 2-7 h post inoculation (2-7h; hatched bars): ammonium chloride (NH_4Cl), Bafilomycin A1 (BafA1), Chloroquine (Chloq), Chlorpromazine (Chlopro), Monensin (Mon), Dynasore, Dyngo-4A (Dyngo), EIPA, Latrunculin A (LatA), Jasplakinolide (Jasp), Cytochalasin B (CytoB), Cytochalasin D (CytoD), Nocodazole (Noc), MG132, Brefeldin A (BrefA), as well as solvents dimethyl sulfoxide (DMSO) and methanol (MeOH). Infection levels were determined by measuring the luciferase activity in cell lysates relative to mock-treated cells (UNTR). **B)** Pan-lysosomal protease inhibitor CPI blocks infection of LR7 cells with MHV-EFLM (MHV-A59) but not with MHV-S2'FCS. Cells were pretreated with increasing concentrations of CPI for 30min. Subsequently cells were inoculated with luciferase-expressing MHV-A59 or MHV-S2'FCS at MOI=0.2. Inoculum was removed at 2hpi and infection allowed to continue until 7hpi. The inhibitor was kept present at the same concentration throughout the experiment. Infection levels were determined by measuring the luciferase activity in cell lysates relative to lysates of mock-treated cells. **C)** U18666A

inhibits infection of LR7 cells with MHV-EFLM (MHV-A59) but not MHV-S2’FCS. Concentration dependent-inhibition of infection by U18666A was determined as described in B for CPI. **A-C)** Error bars represent SEM, n=3*3.

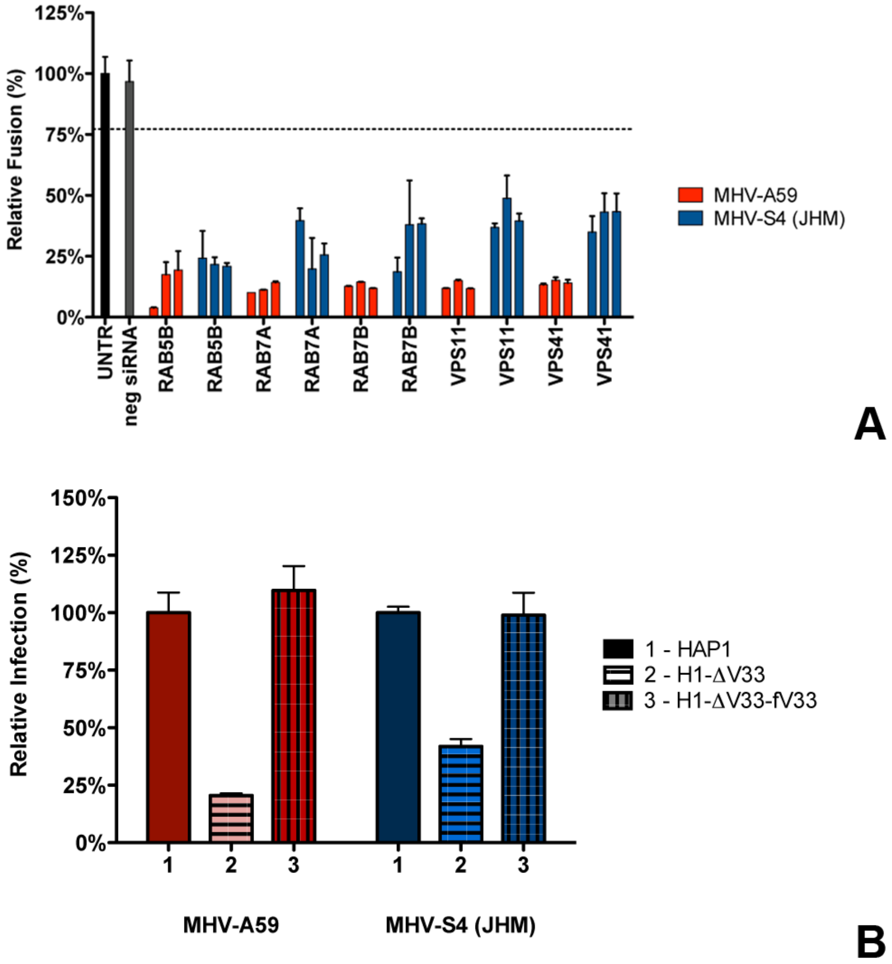

Figure S12. MHV-S4 (JHM) requires late endosomal factors and a functional HOPS complex for efficient infection. A) siRNA-mediated gene silencing was performed as described in the legend to Figure 1. At 72h post transfection, HeLa-mCC1a were inoculated with MHV-EFLM-S4 (JHM) or MHV-EFLM at MOI=0.2 and incubated until 7hpi. Infection levels were determined by measuring the luciferase activity in cell lysates relative to mock-treated cells. Dotted line shows the lower 95% confidence interval of the negative siRNA controls. **B)** Haploid HAP1 cells (HAP1), haploid cells lacking VPS33A (H1-ΔV33) or VPS33A-lacking haploid cells retransfected with FLAG-tagged VLP33A (H1-ΔV33-fV33) were infected (MOI=0.2) with MHV-EFLM (MHV-A59) or MHV-S4 (JHM) for 7h. Infection levels were determined by measuring the luciferase activity in cell lysates relative to mock-treated cells. **A, B)** Error bars represent SEM, n=3*3.
