# Supplementary material for: Correction: Coronavirus Cell Entry Occurs through the Endo-/Lysosomal Pathway in a Proteolysis-Dependent Manner
Source: PLoS Pathog. 2015 Feb 13;11(2):e1004709. doi: 10.1371/journal.ppat.1004709 (PMC4334521; doi:10.1371/journal.ppat.1004709)
Supplement: S2 File — (PDF) [file ppat.1004709.s002.pdf]

Coronavirus Cell Entry Occurs through the Endo-/Lysosomal Pathway in a Proteolysis-Dependent Manner

Christine Burkard[1¤a], Monique H. Verheije[1¤b], Oliver Wicht[1], Sander I. van Kasteren[2], Frank J. van Kuppeveld[1], Bart L. Haagmans[3], Lucas Pelkmans[4], Peter J. M. Rottier[1], Berend Jan Bosch[1], Cornelis A. M. de Haan[1]*

1 Virology Division, Department of Infectious Diseases and Immunology, Faculty of Veterinary Medicine, Utrecht University, Utrecht, The Netherlands, **2** Division of Bio-Organic Synthesis, Leiden Institute of Chemistry, Leiden University, Leiden, The Netherlands, **3** Department of Viroscience, Erasmus MC, Rotterdam, The Netherlands, **4** Institute of Molecular Life Sciences, University of Zurich, Zurich, Switzerland

Abstract

Enveloped viruses need to fuse with a host cell membrane in order to deliver their genome into the host cell. While some viruses fuse with the plasma membrane, many viruses are endocytosed prior to fusion. Specific cues in the endosomal microenvironment induce conformational changes in the viral fusion proteins leading to viral and host membrane fusion. In the present study we investigated the entry of coronaviruses (CoVs). Using siRNA gene silencing, we found that proteins known to be important for late endosomal maturation and endosome-lysosome fusion profoundly promote infection of cells with mouse hepatitis coronavirus (MHV). Using recombinant MHVs expressing reporter genes as well as a novel, replication-independent fusion assay we confirmed the importance of clathrin-mediated endocytosis and demonstrated that trafficking of MHV to lysosomes is required for fusion and productive entry to occur. Nevertheless, MHV was shown to be less sensitive to perturbation of endosomal pH than vesicular stomatitis virus and influenza A virus, which fuse in early and late endosomes, respectively. Our results indicate that entry of MHV depends on proteolytic processing of its fusion protein S by lysosomal proteases. Fusion of MHV was severely inhibited by a pan-lysosomal protease inhibitor, while trafficking of MHV to lysosomes and processing by lysosomal proteases was no longer required when a furin cleavage site was introduced in the S protein immediately upstream of the fusion peptide. Also entry of feline CoV was shown to depend on trafficking to lysosomes and processing by lysosomal proteases. In contrast, MERS-CoV, which contains a minimal furin cleavage site just upstream of the fusion peptide, was negatively affected by inhibition of furin, but not of lysosomal proteases. We conclude that a proteolytic cleavage site in the CoV S protein directly upstream of the fusion peptide is an essential determinant of the intracellular site of fusion.

Citation: Burkard C, Verheije MH, Wicht O, van Kasteren SI, van Kuppeveld FJ, et al. (2014) Coronavirus Cell Entry Occurs through the Endo-/Lysosomal Pathway in a Proteolysis-Dependent Manner. PLoS Pathog 10(11): e1004502. doi:10.1371/journal.ppat.1004502

Editor: Stanley Perlman, University of Iowa, United States of America

Received May 26, 2014; **Accepted** October 2, 2014; **Published** November 6, 2014

Copyright: © 2014 Burkard et al. This is an open-access article distributed under the terms of the Creative Commons Attribution License, which permits unrestricted use, distribution, and reproduction in any medium, provided the original author and source are credited.

Data Availability: The authors confirm that all data underlying the findings are fully available without restriction. All relevant data are within the paper and its Supporting Information files.

Funding: This work was supported by the EU 7th Framework Programme (Virus Entry, project 235649, PJMR) and by a Utrecht University High potential grant to CAMdH. The funders had no role in study design, data collection and analysis, decision to publish, or preparation of the manuscript.

Competing Interests: The authors have declared that no competing interests exist.

* Email: c.a.m.dehaan@uu.nl

¤a Current address: The Roslin Institute and Royal (Dick) School of Veterinary Studies, University of Edinburgh, Easter Bush, Edinburgh, United Kingdom,
¤b Current address: Department of Pathobiology, Division Pathology, Faculty of Veterinary Medicine, Utrecht University, Utrecht, The Netherlands

Introduction

To achieve successful infection enveloped viruses need to fuse with a host cell membrane to deliver the viral genome into the host cell. Some viruses, such as herpes simplex virus, Sendai virus, and human immunodeficiency virus, appear to be capable of direct fusion at the plasma membrane after initial attachment [1–5]. However, the majority of enveloped viruses use endocytosis for uptake and transport prior to fusion. Since endocytic cargo may eventually end up in the destructive environment of the lysosome, environmental cues are crucial to trigger viral fusion at the right stage of trafficking. These triggers, which may include a decrease in pH, changes in redox environment, and proteolytic activity [6–8], induce conformational changes in the viral fusion proteins leading to the merger of viral and host membranes. Two well-studied viruses; influenza A virus (IAV) and vesicular stomatitis virus (VSV), are known to undergo fusion upon exposure to low pH [9–12]. Other enveloped viruses, such as respiratory syncytial virus (RSV) and Ebola virus, require proteolytic processing of their viral fusion proteins in the endosomal system for fusion to occur [13–16].

Coronaviruses (CoVs) are enveloped, plus-strand RNA viruses belonging to the family *Coronaviridae* in the order *Nidovirales*. They are capable of infecting a wide variety of mammalian and avian species. In most cases they cause respiratory and/or intestinal tract disease. Human coronaviruses (HCoVs) are known as major causes of the common cold (e.g. HCoV-229E and HCoV-OC43). However, the emergence of new HCoVs of zoonotic origin has shown the potential of CoVs to cause life-threatening disease in humans as was demonstrated during the 2002/2003 SARS-CoV epidemics and more recently for MERS-CoV in the Middle East [17,18]. The well-studied mouse hepatitis virus (MHV) is often used as a safe model to study CoV infections.

Author Summary

Enveloped viruses need to fuse with a host cell membrane in order to deliver their genome into the host cell. In the present study we investigated the entry of coronaviruses (CoVs). CoVs are important pathogens of animals and man with high zoonotic potential as demonstrated by the emergence of SARS- and MERS-CoVs. Previous studies resulted in apparently conflicting results with respect to CoV cell entry, particularly regarding the fusion-activating requirements of the CoV S protein. By combining cell-biological, infection, and fusion assays we demonstrated that murine hepatitis virus (MHV), a prototypic member of the CoV family, enters cells via clathrin-mediated endocytosis. Moreover, although MHV does not depend on a low pH for fusion, the virus was shown to rely on trafficking to lysosomes for proteolytic cleavage of its spike (S) protein and membrane fusion to occur. Based on these results we predicted and subsequently demonstrated that MERS- and feline CoV require cleavage by different proteases and escape the endo/lysosomal system from different compartments. In conclusion, we elucidated the MHV entry pathway in detail and demonstrate that a proteolytic cleavage site in the S protein of different CoVs is an essential determinant of the intracellular site of fusion.

All CoV virions contain a canonical set of four structural proteins. The viral genomic RNA is encapsidated by the nucleocapsid protein (N) to form the helical nucleocapsid, which is surrounded by the lipoprotein envelope, containing membrane glycoprotein (M), the small envelope protein (E), as well as the spike glycoprotein (S) (reviewed in [19]). Trimers of the CoV S protein, a type I membrane protein belonging to the class I fusion proteins, form the peplomers that protrude from the virion surface [20]. The S protein can be divided into two functional subunits. The amino-terminal S1 subunit contains the receptor-binding domain; while the carboxy-terminal S2 subunit contains domains required for fusion, including the fusion peptide (FP), heptad repeat domains (HR) HR1 and HR2, and the transmembrane (TM) domain.

Various entry routes have been described as being used by different CoVs for infection of cells. Clathrin-dependent as well as clathrin- and caveolae-independent entry pathways have been reported for SARS-CoV [21,22]. Also feline infectious peritonitis virus (FIPV) was suggested to enter via a clathrin- and caveolae-independent endocytic route [23,24]. For the HCoV-229E a caveolae-dependent endocytic uptake has been suggested [25]. Although the ability of MHV S proteins to cause cell-cell fusion at a neutral pH was initially interpreted as an indication for fusion of virions at the cell surface, more recent studies indicate the requirement for clathrin-mediated endocytosis for entry of MHV [26–29]. However, while some studies report that MHV strain A59 is sensitive to lysosomotropic agents that affect endocytosis [26], this is not the case according to others [27].

Proteolytic cleavage of the CoV S proteins appears to be important for the induction of cell-cell fusion and/or virus entry into host cells. Different cleavage sites have been identified for different CoVs, the importance of which seems to differ for cell-cell and virus-cell fusion. Some CoV S proteins, including that of MHV strain A59, are cleaved at the S1/S2 boundary by furin(-like) proteases during transport of the newly assembled virions through the secretory pathway of the producer cell [30–33]. Inhibition of this S protein cleavage was shown to inhibit cell-cell fusion, but not to affect entry of MHV strain A59 into host cells [30,34,35]. MHV strain 2 contains an S protein that is not cleaved at the S1/S2 boundary. Interestingly, although MHV strains 2 and A59 were both reported to enter via clathrin-mediated endocytosis, entry of MHV 2 but not of MHV A59, was blocked by inhibitors of low-pH activated cathepsin proteases [27,36]. Inhibitors of cathepsin proteases have also been shown to inhibit entry of SARS-CoV and feline CoVs [23,37,38], while treatment of cell-bound virus particles with different proteases was shown to enhance virus entry and/or cell-cell fusion [27,34,39–45]. For SARS-CoV and infectious bronchitis virus (IBV), it appears that a proteolytic cleavage of the S protein at a more downstream position than the S1/S2 boundary upon receptor binding is of importance for cell entry [40,43,46–49].

In the present study we performed a detailed investigation of the entry of different CoVs. Using siRNA gene silencing, we found that the prototypic coronavirus MHV strain A59 (further referred to as MHV) requires proteins known to be important for late endosomal maturation and endosome-lysosome fusion for efficient infection of cells. By using recombinant MHVs expressing reporter genes as well as by applying a novel, replication-independent fusion assay we confirmed the importance of clathrin-mediated endocytosis and demonstrated that trafficking of MHV virions to lysosomal compartments and processing of the S protein by lysosomal proteases was required for productive entry to occur. Our results indicate that a cleavage site in the S protein of CoVs immediately upstream of the FP determines the site of fusion. In agreement herewith FIPV, which requires processing by lysosomal proteases, was also shown to depend on trafficking to lysosomes. In contrast, MERS-CoV, which contains a minimal furin-cleavage site consensus sequence in the S protein immediately upstream of the FP, was negatively affected by inhibition of furin, but not of lysosomal proteases.

Results

RNAi mediated gene silencing identifies endocytosis-associated proteins to be important in MHV infection

In an automated, high-throughput RNAi screen [50] targeting the druggable genome (approximately 7000 genes) a number of proteins associated with endocytosis were found to be required for efficient infection of HeLa cells with GFP-expressing MHV. To validate these findings these proteins were subjected to a follow-up analysis using siRNA-mediated gene silencing with oligonucleotides from a different supplier than the one used for the initial RNAi screen (Fig. 1A). The follow-up analysis included ACTR2 and ACTR3, two major constituents of the Arp2/3 complex which are important for the formation of actin branches and cell surface protrusions, as well as for the motility of several pathogens inside host cells (reviewed in [51,52]). Also selected were the RAS-related GTP-binding protein family members, RAB7A and RAB7B, which have been shown to be involved in endosomal maturation (reviewed in [53]). RAB7 interacts amongst others with members of the homotypic fusion and vacuole protein sorting (HOPS) tethering complex, involved in late endosome to lysosome maturation. The HOPS subunit VPS39 (reviewed in [54]) was also found to be a strong hit in the siRNA screen and therefore selected. Other proteins included SNX1, involved in retrograde transport of cargo between endosomes and the *trans*-Golgi network (reviewed in [55]), VCL, inter alia involved in connecting the Arp2/3 complex with integrins during actin polymerization (reviewed in [56]), and the Ser/Thr-protein kinase PAK1, which is activated by the Rho/Rac/Cdc42 family and is implicated in a variety of downstream effects including modulation of the actin cytoskeleton (reviewed in [57]).

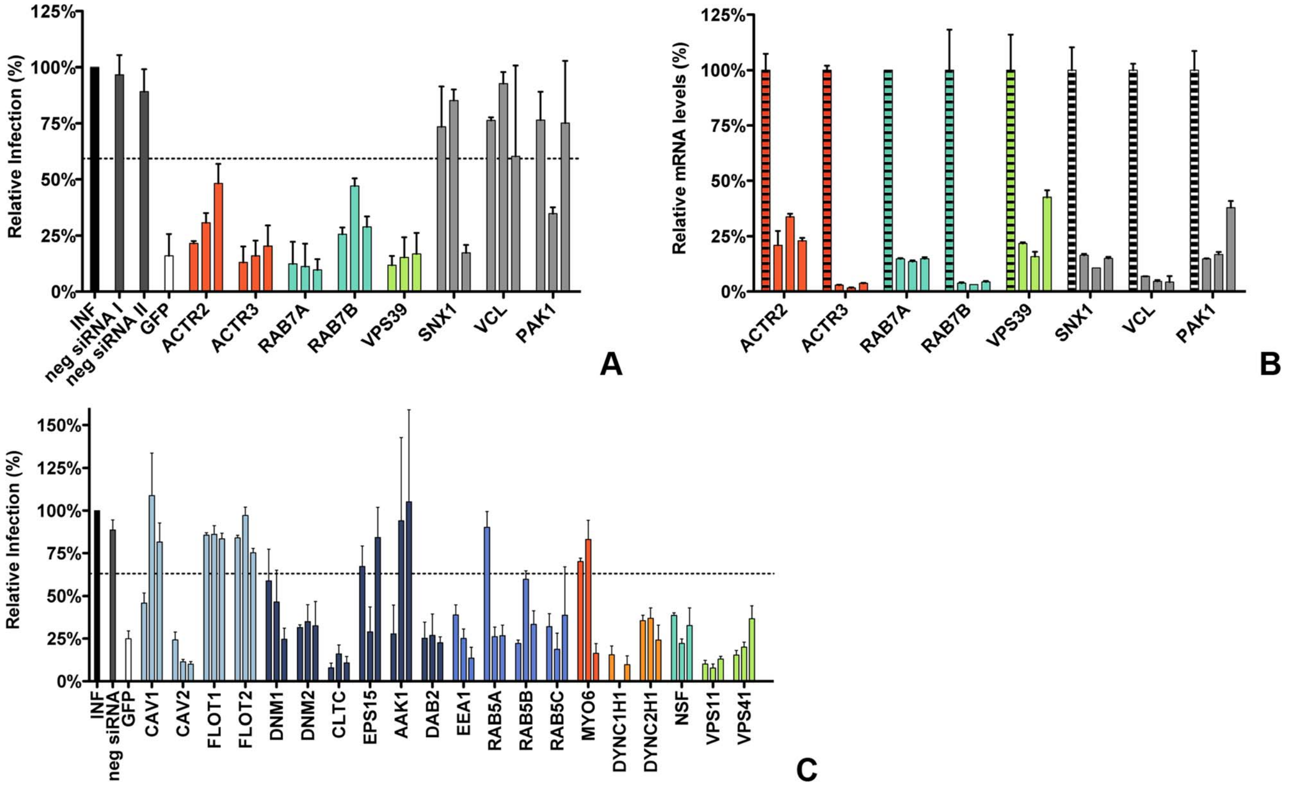

Figure 1. RNAi-mediated downregulation of endocytosis-associated proteins affects MHV infection. A) Confirmation of endocytosis-associated hits from druggable genome-wide siRNA screen. Gene silencing was performed using individual transfection of three different siRNAs per gene in HeLa-mCC1a cells. Cells were infected with MHV-EGFPM at MOI = 0.5 for 8 h and analyzed by FACS for cell viability and virus replication. The effect of downregulation of expression on MHV infection was studied for the actin cytoskeleton-associated proteins ACTR2 and ACTR3 (orange), late endosomal proteins RAB7A and RAB7B (turquoise), HOPS complex sububit VPS39 (light green), ER/Golgi secretion-associated protein SNX1, Integrin/Actin-associated protein VCL, and Serine/Threonine-protein kinase PAK1 (grey). Error bars represent SEM, n = 4. **B**) Confirmation of siRNA-mediated reduction in mRNA levels. mRNA levels at 72 h post transfection were measured by qRT-PCR in comparison to non-transfected cells. Error bars represent SEM, n = 3*3. **C**) The effect of the RNAi-mediated downregulation of an extended set of endocytosis-associated proteins on MHV infection. Infection of MHV-EGFPM was analyzed after downregulation of proteins associated with caveolae-mediated endocytosis (light blue), clathrin-mediated endocytosis (dark blue), early endosomes (cerulean), actin cytoskeleton (dark orange), microtubule cytoskeleton (orange), late endosomes (turquoise), and late endosome-to-lysosome trafficking (light green) as described above. Error bars represent SEM, n = 3. **A, C**) Dotted lines show the lower 95% confidence interval of the negative siRNA controls.
doi:10.1371/journal.ppat.1004502.g001

Transfection of HeLa cells carrying the receptor for MHV (HeLa-mCC1a cells) with different siRNAs was followed by an infection with GFP-expressing MHV (MHV-EGFPM) at low multiplicity of infection (MOI), resulting in approximately 10–15% infected cells under control conditions. After 8 h of infection cells were collected and GFP expression by the replication of MHV was analyzed by fluorescence-activated cell sorting (FACS). As controls siRNAs silencing GFP and negative-control siRNAs were used. A hit from the screen was considered as confirmed when transfection with at least two out three independent siRNAs resulted in significant reduction in MHV-driven GFP expression relative to the negative-control siRNAs. siRNA-mediated gene silencing of ACTR2 and ACTR3 resulted in reduced infections for all three siRNAs, indicating that actin branching is important for MHV infection (Figure 1A, dark orange). Also the importance of the RAB7A, RAB7B and VPS39 proteins, involved in late-endosome and late-endosome to lysosome maturation, for MHV infection could be confirmed (Figure 1A, turquoise and light green). The importance of SNX1, VCL and PAK1 for infection of HeLa cells with MHV could not be confirmed (Figure 1A, grey). The latter three genes were not studied any further. To validate our transfection protocol and confirm the efficacies of the siRNAs at the mRNA level, quantitative RT-PCR analysis was performed. All siRNAs used reduced the corresponding mRNA levels with 75–95% (Figure 1B). siRNAs targeting RAB7A were shown to inhibit the expression of a RAB7a-fusion protein (Figure S1 in Text S1).

To confirm and extend our understanding of the role of endocytosis in MHV entry we subsequently selected a number of proteins known to be involved in either caveolae- or clathrin-mediated endocytosis, actin- or microtubule-mediated transport, as well as proteins associated with endosomal vesicles and endosomal maturation, to be screened using the siRNA silencing-approach described above. Again, proteins were considered important for infection with MHV when transfection with at least two out three independent siRNAs resulted in significant reduction in MHV-driven GFP expression relative to the negative-control siRNAs. siRNA-mediated downregulation of proteins involved in caveolae-mediated endocytosis revealed that CAV2, but not the other proteins analyzed are important for infection with MHV (Figure 1C, light blue). Downregulation of most proteins associated with clathrin-mediated endocytosis inhibited MHV infection,

including DNM1, DNM2, CLTC, and DAB2. siRNA-mediated silencing of EPS15 or AAK1, accessory factors of clathrin-mediated endocytosis, did not affect MHV replication (Figure 1C, dark blue). Silencing of early endosome-associated genes (EEA1, RAB5A, RAB5B, and RAB5C; Figure 1C, cerulean) each decreased replication-mediated GFP expression. While downregulation of MYO6, involved in actin-based motility, did not influence MHV infection (Figure 1C, dark orange), our results indicate that the microtubule-associated motility proteins DYNC1H1 and DYNC2H1 are important for infection with MHV (Figure 1C, orange). Silencing of NSF, required for transport from early to late endosomes [58], or of the HOPS subunits VPS11 and VPS41, which are involved in late endosome to lysosome maturation (Reviewed in [54]), all resulted in severely reduced MHV infection (Figure 1C, turquoise and light green, respectively).

Endocytosis-affecting agents indicate clathrin-mediated endocytosis and endosome maturation to be important in MHV infection

To further explore the endocytic route and factors involved in MHV infection we determined the effect of inhibitors on MHV infection. HeLa-mCC1a cells were treated with endocytosis-affecting agents for 30 min and then infected with luciferase-expressing MHV (MHV-EFLM; [59]) in presence of the inhibitors, after which the inhibitors were kept present until cell lysis. When cells were inoculated with MHV-EFLM in the absence of inhibitors, the inhibitors were added to the cells at 2 h post infection (hpi) to assess effects of inhibitors on post-entry steps. At 7 hpi cells were lysed and firefly luciferase expression levels were determined.

Infection in the presence of the solvents dimethyl sulfoxide (DMSO) and methanol (MeOH), as well as the known inhibitors of MHV RNA synthesis Brefeldin A (BrefA, inhibitor of GBF1) [60] and MG132 (proteasome inhibitor, probably also affects MHV entry; [61]) were included as controls. MHV infection was not affected by addition of the solvents, whereas both MG132 and BrefA severely decreased luciferase expression regardless of the time of addition. Inhibition of endosome maturation with ammonium chloride (NH_4Cl), Bafilomycin A1 (BafA1), or Chloroquine (Chloq) severely diminished luciferase expression when the inhibitors were added prior to infection. Much smaller effects were observed when these drugs were added at 2 hpi, indicating that the inhibitors mainly affect MHV entry (Figure 2, deep sky blue). Similar effects were observed with known inhibitors of clathrin-mediated endocytosis; Chlorpromazine (Chlopro), Monensin (Mon), Dynasore, and Dyngo-4A (Dyngo). All these compounds strongly decreased MHV replication-mediated luciferase expression when added early but not when added at 2 hpi (Figure 2, dark blue). The actin- and macropinocytosis-affecting drug EIPA, which inhibits the Na^+/H^+ exchanger NHE1, led to reduced luciferase expression both when added prior to and after entry of MHV at 2 hpi. Actin cytoskeleton altering drugs Latrunculin A (LatA), Jasplakinolide (Jasp), Cytochalasin B (CytoB), and Cytochalasin D (CytoD), or the inducer of microtubule depolymerization Nocodazole (Noc) only decreased MHV infection when added early, indicating a role for the actin and microtubule cytoskeleton in entry but not RNA replication (Figure 2, dark orange and orange). Likewise U18666A, a cholesterol transport-affecting agent, which also prevents maturation of late endosomes [62], had a strong inhibitory effect on MHV infection when added early (Figure 2, turquoise). Collectively, these results indicate an important role for clathrin-mediated uptake and for endosome- and endosome-to-lysosome maturation for MHV infection.

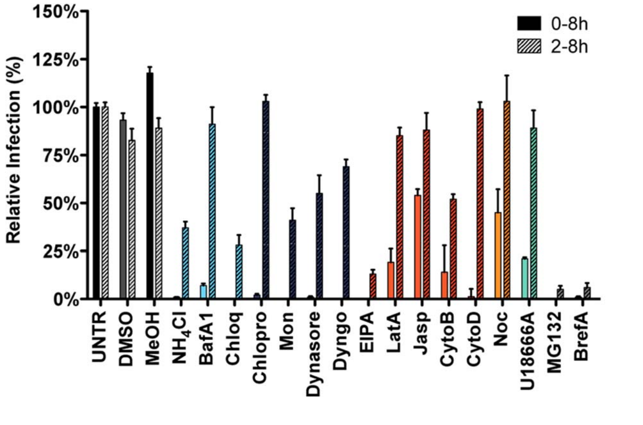

Figure 2. Endocytosis affecting agents indicate clathrin-mediated endocytosis and endosome maturation to be important in MHV infection. HeLa-mCC1a cells, inoculated with MHV-EGFPM at MOI = 0.5, were treated with the different inhibitors from 30 min prior to 8 h post inoculation (0–8 h) or from 2–8 h post inoculation (2–8 h; hatched bars): ammonium chloride (NH_4Cl), Bafilomycin A1 (BafA1), Chloroquine (Chloq), Chlorpromazine (Chlopro), Monensin (Mon), Dynasore, Dyngo-4A, EIPA, Latrunculin A (LatA), Jasplakinolide (Jasp), Cytochalasin B (CytoB), Cytochalasin D (DytoD), Nocodazole (Noc), MG132, Brefeldin A (BrefA), as well as solvents dimethyl sulfoxide (DMSO) and methanol (MeOH). Infection was determined by FACS and displayed relative to the infection level observed in mock-treated cells (UNTR). Error bars represent SEM, n = 3.
doi:10.1371/journal.ppat.1004502.g002

Clathrin-mediated endocytosis and late endosomal factors are required for MHV fusion

The time-of-addition experiments with the different inhibitors indicated that particularly the entry step of the MHV infection cycle is negatively affected by perturbation of clathrin-mediated endocytosis or of endosome maturation. However, assays based on reporter gene expression driven by virus replication do not allow discrimination between virus entry and RNA replication when analyzing siRNAs or agents that also affect RNA synthesis. To unequivocally demonstrate the importance of clathrin-mediated endocytosis and endosome maturation for MHV entry, we therefore made use of a fusion assay we recently developed [63]. The assay is based on minimal complementation of defective β-galactosidase (β-galactosidase ΔM15) with the short α-peptide [64]. MHV-αN, a recombinant MHV containing an N protein tagged with the α-peptide (αN), is used to infect ΔM15−fragment expressing target cells. Upon fusion of the virion with a host cell membrane αN is released into the cytoplasm resulting in complementation of the defective β-galactosidase thereby reconstituting a functional enzyme. Conversion of the non-fluorescent substrate fluorescein-di-β-D-galactopyranoside (FDG) by β-galactosidase into green fluorophores fluorescein (FIC) can be measured by FACS or fluorescence microscopy (Figure S2 in Text S1).

To analyze the effect of RNAi-mediated gene silencing on fusion, HeLa cells expressing the MHV receptor and the ΔM15−fragment (HeLa-mCC1a-ΔM15 cells) were transfected with individual siRNAs and inoculated with MHV-αN at 72 h post transfection. Before infection cells were pre-loaded with FDG by hypotonic shock. After 100 min incubation of cells with virus at 37°C, cells were collected and the amount of FIC generated as a results of enzyme complementation analyzed by FACS. The fusion assay showed that silencing of neither CAV1 nor CAV2 affected MHV fusion (Figure 3A, light blue), even though reduction of CAV2 was shown to affect MHV infection (Figure 1C). However, downregulation of clathrin-mediated endocytosis associated pro-

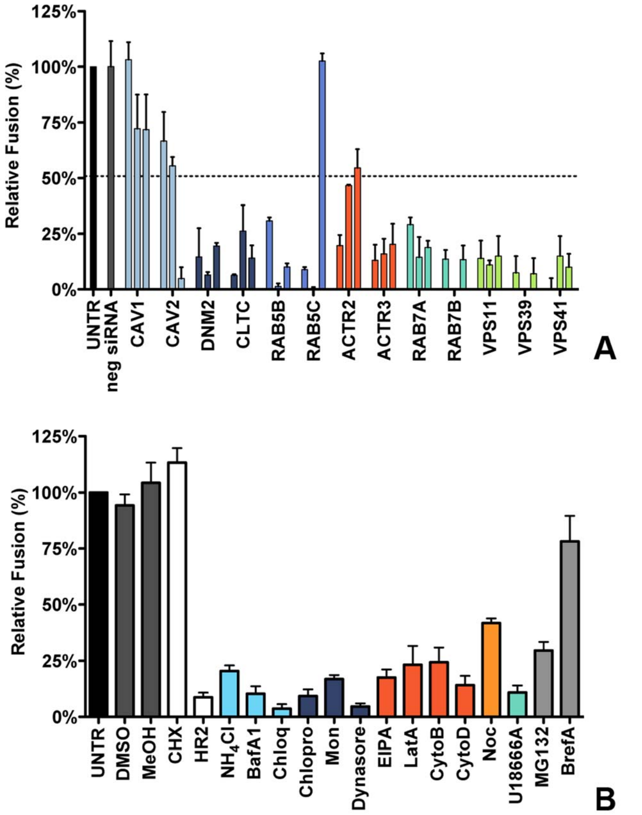

Figure 3. Clathrin-mediated endocytosis and late endosome-to-lysosome trafficking is required for MHV fusion. A) Fusion assay upon siRNA-mediated gene silencing. Three different siRNAs per gene were transfected individually into HeLa-mCC1a-ΔM15. 72 h post transfection, cells were pre-loaded with FDG by hypotonic shock. MHV-αN was allowed to bind to the cells on ice at MOI = 20 for 90 min. 100 min post warming to 37°C, cells were collected and analyzed by FACS. Fusion was determined relative to the number of FIC-positive cells observed upon mock treatment of infected cells (UNTR). Error bars represent SEM, n = 3. **B**) Fusion of MHV upon treatment of cells with different inhibitors was studied as in A. Cells were pretreated with ammonium chloride (NH4Cl), Bafilomycin A1 (BafA1), Chloroquine (Chloq), Chlorpromazine (Chlopro), Monensin (Mon), Dynasore, Dyngo-4A, EIPA, Latrunculin A, (LatA), Jasplakinolide (Jasp), Cytochalasin B (CytoB), Cytochalasin D (DytoD), Nocodazole (Noc), U18666A, MG132, Brefelding A (BrefA), as well as with the solvents dimethyl sulfoxide (DMSO) and methanol (MeOH), protein synthesis inhibitor cyclohexamide (CHX), and MHV fusion inhibitor HR2 peptide (HR2) for 30 min at 37°C. The inhibitors were kept present during binding of MHV-αN to cells and during warming to 37°C cells for 100 min. Fusion was determined relative to the number of FIC-positive cells after mock treatment (UNTR). Error bars represent SEM, n = 3.
doi:10.1371/journal.ppat.1004502.g003

teins DNM2 and CLTC lead to strongly decreased fusion, as did the lack of early endosome-associated factors RAB5B and RAB5C (Figure 3A, dark blue and cerulean, respectively). Fusion was also affected by RNAi-mediated reduction of actin cytoskeleton-associated proteins ACTR2 and ACTR3 (Figure 3A, dark orange), proteins known to be involved in late endosome (RAB7A, RAB7B) and late endosome-to-lysosome maturation (VPS11, VPS39, and VPS41) (Figure 3A, turquoise and light green).

The importance of clathrin-mediated endocytosis and endosome maturation for MHV fusion was confirmed by analysis of endocytosis-affecting agents using the fusion assay. After pre-loading with FDG, cells were pre-treated with the inhibitors for 30 min at 37°C, after which cells were inoculated with MHV-αN in the presence of the agents, and analyzed by FACS as described above. As controls we included protein synthesis inhibitor cycloheximide (CHX), MHV fusion inhibitor peptide HR2 (HR2, [20]), MG132 and BrefA. Fusion of MHV was not affected by the solvents or CHX, the latter confirming that this assay is independent of RNA replication and protein synthesis. MHV fusion was barely affected by replication inhibitor BrefA, whereas MG132 had a clear negative effect, in agreement with the conclusion drawn previously that MG132 inhibits entry of MHV as well as RNA synthesis [61]. Inhibition of endosomal maturation by NH_4Cl, BafA1 and Chloq (Figure 3B, deep sky blue) or of clathrin-mediated endocytosis by Chlopro, Mon, and Dynasore (Figure 3B, dark blue) severely inhibited MHV fusion. Disturbance of the actin cytoskeleton by EIPA or by LatA, CytoB, or CytoD reduced fusion by 75–80% (Figure 3B, dark orange), while interference with microtubule polymerization by Noc had a smaller effect (Figure 3B, orange). Late endosomal maturation arrest caused by U18666A reduced fusion to approximately 10% (Figure 3B, turquoise). In conclusion, the replication-independent fusion assay confirmed the importance of clathrin-mediated endocytosis and of endosome maturation for entry of MHV. The data indicate that late endosome-to-lysosome maturation is required for efficient entry and fusion.

Live-cell microscopy confirms co-localization, co-tracking and fusion of MHV in endosomal compartments

To confirm the importance of endocytic uptake and the association of MHV with endosomal compartments we performed live-cell confocal microscopy. To this end, sucrose density gradient-purified MHV virus was covalently labeled with the low-pH resistant dye DyLight 488 (MHV-DL488). HeLa-mCC1a cells were transfected with plasmids to express monomeric RFP (mRFP) fusion proteins of RAB5, RAB7, or LAMP1. At 24 h post transfection, MHV-DL488 was bound to cells at 4°C for 90 min. Inoculation medium was replaced by warm medium containing trypan blue, which immediately shifts the emission spectrum of surface bound particles rendering them undetectable in the 505–530 nm channel unless they get endocytosed [65]. Cells were imaged using a spinning-disc confocal microscope acquiring z-stacks in 30 s intervals over 10 min time frames from 10–70 min post warming. Only low-level RFP fusion protein expressing cells were selected for analysis. Interestingly, MHV particles newly appeared even 60 min post warming, in agreement with the notion that MHV enters in an unsynchronized manner (unpublished results). Co-localization and co-trafficking of viruses with endosomal compartments was assessed by detecting virus particles based on size and intensity (green channel) and by measuring the underlying intensity in the red channel (endosomal vesicles). MHV virions were found to co-localize with all three endosomal compartments (Fig. 4A). Whereas newly entering/appearing particles were always co-localizing with RAB5 molecules, they only associated with RAB7 and LAMP1 containing vesicles at later time points.

To assess the association of MHV with endosomal vesicles during the entry process more extensively, we manually tracked the virus particles in the green channel and independently tracked the endosomal vesicles in the red channel in x/y and z-direction. A virion was categorized as associating with a certain endosomal marker only if this co-localization was observed over at least four sequential 30 s interval images. When the initial co-localization was lost, but the virion did not disappear, this virion was classified as associating/dissociating. Complete disappearance of a virus particle (including in other z-stacks) while immediately previously co-localizing with an endosomal marker was categorized as a

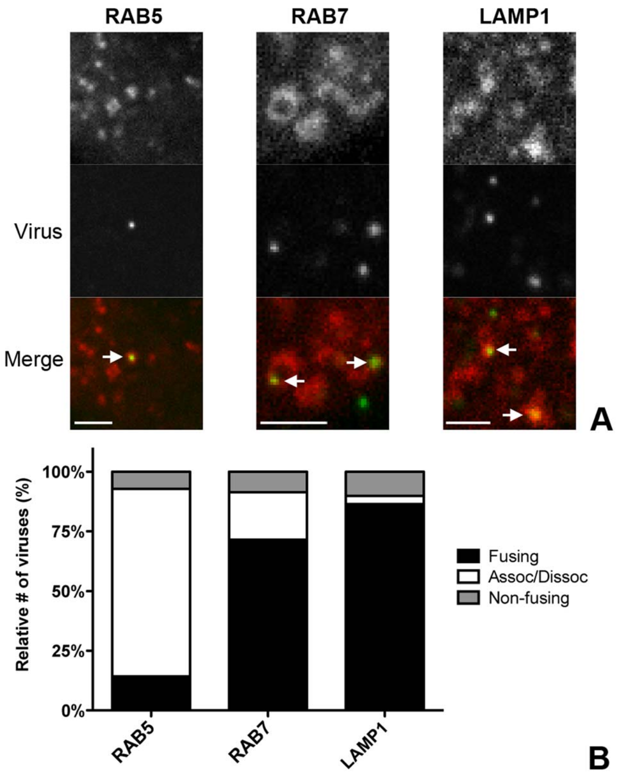

Figure 4. Live-cell microscopy demonstrates co-localization and co-tracking of MHV with endosomal vesicles and fusion of MHV in these vesicles. HeLa-mCC1a cells transfected with plasmids encoding RAB5-mRFP, RAB7-mRFP, or dsRed-LAMP1 were inoculated with DyLight 488-labeled MHV. Live cell imaging was performed to track internalized particles. **A**) Examples of MHV particles co-localizing with RAB5-, RAB7-, and LAMP1-positive endosomal vesicles. Size bars indicate 0.2 μM **B**) Virus particles that could be tracked were classified as 'fusing' (Fusing) 'associating/dissociating' (Assoc/Dissoc), or 'non-fusing' (Non-fusing) as described in the Materials and Methods section.
doi:10.1371/journal.ppat.1004502.g004

fusion event (Figures S3 and S4 in Text S1). When a viral particle co-localized with endosomal compartments but did neither dissociate nor fade during the 10 min acquisition period it was classified as non-fusing. With this quantification method we analyzed 75–100 virions in total for each of the endosomal compartment types studied. The fraction of virions not fusing during the acquisition period was consistently found to be at around 10–15%. We observed that all of the entering MHV particles initially co-localized with RAB5-positive early endosomal vesicles and that most virions dissociated (were no longer co-localized) after 4–6 min. Notably, it appeared that in these events the RAB5 marker faded rather than moved away. Only a very small percentage of virions were categorized as fusing while in early endosomes. The number of fusion events was much higher for virions co-localizing with RAB7 or LAMP1 (Figure 4B), indicating that most virions fuse in late endosomes or lysosomes.

MHV infection depends on endosomal maturation

Our results so far indicate that most virions enter cells after having accessed late endosomes/lysosomes. We hypothesized that these compartments provide the environmental cues required for productive virus-cell fusion. In order to analyze to what extent the low pH in the endosomal system is required for entry of MHV, we analyzed the inhibition of MHV entry at different concentrations of BafA1. While high concentrations of BafA1 (as used for the results shown in Fig. 2 and 3) affect endosomal maturation, at low concentrations this inhibitor of vacuolar-type H^{+}-ATPase only elevates the pH of endosomal compartments but does not affect endosomal trafficking *per se* [66]. We made use of that property and tested the sensitivity of MHV to BafA1 side by side with the control viruses VSV and IAV. VSV has been described to fuse at pH 6.2 in early and/or late endosomes [9,11,12,67–69], while IAV has been shown to fuse in late endosomes at an even lower pH [9,10,70]. HeLa or HeLa-mCC1a cells were pretreated with increasing concentrations of BafA1 for 30 min prior to infection with reporter gene expressing viruses: VSV (VSVΔG/FLuc-G*; [71,72]), IAV (IAV-RLuc; [73]), or MHV (MHV-EFLM). Luciferase expression levels indicated that infection of cells with VSV and IAV is much more affected by BafA1, with an IC50 values of 0.80 and 0.63 nM, respectively, compared to MHV, which displays a three to four fold higher IC50 of 2.34 nM (Figure 5A).

Our results thus indicate that MHV is much less affected by perturbation of the endosomal pH than VSV and IAV. Nevertheless RNAi-mediated silencing of HOPS subunits and treatment of cells with U1866A indicates that late endosome-to-lysosome maturation is required for efficient entry. To confirm

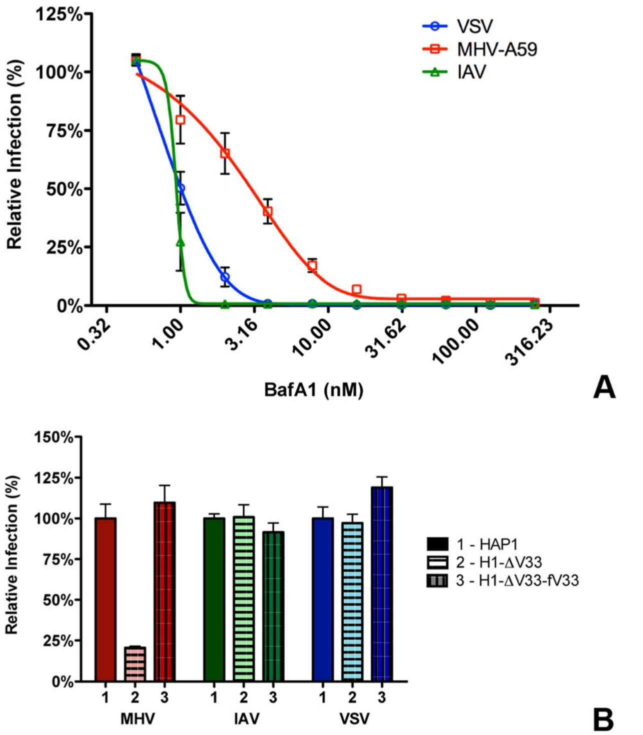

Figure 5. MHV infection depends on endosomal maturation. A) HeLa-mCC1a cells were pretreated with increasing concentrations of Bafilomycin A1 (BafA1) for 30 min and subsequently infected with luciferase expressing MHV, VSV, or IAV in the presence of BafA1. Infection levels were determined by assaying the luciferase activity in cell lysates relative to lysates of infected cells that had been mock treated. Error bars represent SEM, n = 3*3. **B**) Haploid cells (HAP1), haploid cells lacking VPS33A (H1-ΔV33) or VPS33A-lacking haploid cells retransfected with FLAG-tagged VLP33A (H1-ΔV33-fV33) were infected with luciferase expressing MHV, VSV, or IAV. Cells were lysed at 7 h (MHV and VSV) or 16 h post infection. Infection is displayed relative to virus-driven luciferase expression levels in HAP1 cells. Error bars represent SEM, n = 3*3.
doi:10.1371/journal.ppat.1004502.g005

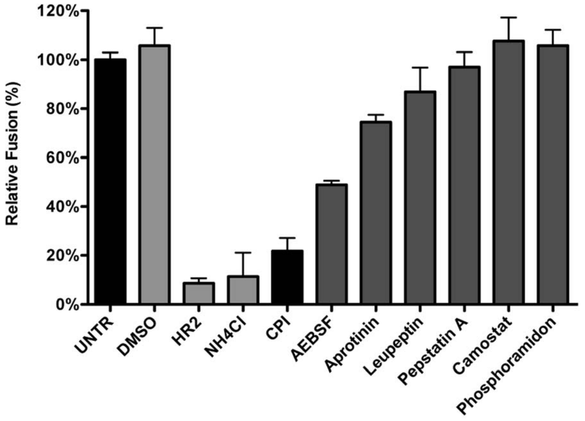

Figure 6. Inhibition of lysosomal proteases prevents MHV fusion. The MHV fusion assay was performed on HeLa-mCC1a-ΔM15 cells as described in the legend to Figure 3, in the presence of the protease inhibitors CPI, AEBSF, Aprotinin, Leupeptin, Pepstatin A, Camostat, and Phosphoramidon. As controls, cells were treated with solvent DMSO, MHV fusion inhibitor HR2 peptide (HR2), and lysosomotropic agent ammonium chloride (NH4Cl). Fusion was determined relative to the number of FIC-positive cells after mock treatment (UNTR). Error bars represent SEM, n = 3.
doi:10.1371/journal.ppat.1004502.g006

and extend these observations, we made use of haploid HAP1 cells lacking a functional HOPS complex resulting from lentiviral-mediated knockout of the VPS33A subunit (H1-ΔV33 cells; [74]).Both HAP1 cells and H1-ΔV33 cells were modified to stably express the MHV receptor. As a control, the H1-ΔV33 cells were in addition stably transfected with FLAG-tagged VPS33A (H1-ΔV33-fV33). The different cells expressed similar levels of the MHV receptor as determined by FACS analysis (Figure S5 in Text S1). Expression of FLAG-VPS33A was confirmed by Western blot (Figure S6 in Text S1). Functional reconstitution was confirmed by confocal fluorescence imaging of lysosome localization (Figure S7 in Text S1). While in the knockout cells the lysosomes were clustered, the lysosomes were dispersed again throughout the cytoplasm in the FLAG-VPS33A re-transfected cells, as observed in the HAP1 parental cells. The haploid cells were infected with luciferase reporter gene-expressing MHV, VSV, or IAV at low MOI. Cells were lysed at 7 (MHV and VSV) or 16 (IAV) hpi and luciferase expression levels were determined. The lack of a functional HOPS complex had no effect on VSV and IAV infection; however, MHV infection was strongly reduced in the knockout, but not in the re-transfected cells (Figure 5B). These observations confirm the conclusion that late endosome-to-lysosome maturation is required for efficient entry of MHV, a characteristic that is not shared with the pH-sensitive VSV and IAV.

Inhibition of lysosomal proteases prevents MHV fusion

Considering that MHV was much less affected by perturbation of the endosomal pH than IAV and VSV while it requires trafficking to lysosomes for efficient entry, we hypothesized that entry might depend on cleavage of a viral protein by lysosomal proteases. Hence we analyzed the extent to which different protease inhibitors could inhibit MHV entry. Thus, HeLa-mCC1a-ΔM15 cells were pretreated for 30 min with the different inhibitors, after which the cells were inoculated with MHV-αN in inhibitor-containing medium. Cells were collected, loaded with FDG, and FDG conversion to FIC by complementation of β-galactosidase upon viral fusion was assessed by FACS. Our results indicate that most protease inhibitors tested (Fig. 6) hardly inhibited fusion of MHV, if at all. Exceptions were AEBSF, which has been shown to cause aggregation of early endosomal vesicles [75], and a pan-lysosomal protease inhibitor (CPI; cystatin-pepstatin inhibitor) capable of inhibiting the three major protease family members found in lysosomes. Thus, by using CPI we measured the combined effects of an endosomal papain-like cysteine protease inhibitor (PLCP), an aspartyl protease inhibitor, and an asparagine endopeptidase inhibitor (AEP) [76]. From these results we conclude that inhibition of a broad range of endosomal proteases efficiently blocks fusion of MHV, indicating that efficient entry requires the activity of lysosomal proteases.

Introduction of a furin cleavage site immediately upstream of the fusion peptide renders MHV independent of lysosomal proteases

In general, class I fusion proteins require cleavage just upstream of the FP to render them fusion competent [20,38,77]. However, while the S protein of MHV is cleaved at the S1/S2 boundary (Fig. 7A), no protease cleavage site has been identified close to the fusion peptide. In view of the inhibition of MHV entry by the pan-lysosomal protease inhibitor CPI and in analogy to other class I fusion proteins, we hypothesized that an additional cleavage in the S protein, immediately upstream of the FP, is necessary to induce fusion. To test this hypothesis, we introduced an optimal furin cleavage site (FCS) by substituting three amino acids by Arg (AIRG**R**→RRRR**R**) immediately upstream of a highly conserved Arg (indicated in bold) that occurs just N-terminal of the FP. Recombinant MHV carrying this FCS in its S2 subunit was designated MHV-S2′FCS. (Figure 7A). Western blot analysis of the S protein of a purified stock of this virus using an antibody recognizing the S2 subunit showed no evidence of cleavage at the newly introduced FCS (S2′ site). Apparently, cleavage at this position does not occur during virus production (Figure S8 in Text S1). MHV carrying wild type or mutant S proteins displayed similar growth kinetics (Figure S9 A and B in Text S1). Next we analyzed whether the introduced FCS affected the sensitivity of the recombinant MHV to CPI, which does not exhibit inhibitory activity towards furin. Thus, HeLa-mCC1a cells were pretreated with CPI for 30 min and subsequently infected with wild type S (MHV-EFLM) or mutant S (MHV-S2′FCS) containing viruses expressing luciferase reporter genes in the presence of the protease inhibitor. At 7 hpi the cells were lysed and viral-replication dependent luciferase expression levels were determined. Introduction of the FCS resulted in the recombinant virus being no longer sensitive to inhibition by lysosomal proteases (Figure 7B), probably because the S protein is now cleaved by furin in an endocytic compartment.

Furin inhibitor renders MHV-S2′FCS sensitive to endosomal maturation and decreases infection

To confirm that MHV-S2′FCS is no longer dependent on cleavage by lysosomal proteases, and to study its presumed dependence on furin cleavage for entry, we analyzed the ability of MHV-S2′FCS to infect the haploid cells that lack VPS33A - and thus the functional HOPS complex required for late endosome-to-lysosome maturation - in the absence or presence of furin inhibitor (FI). After pretreatment of MHV receptor-expressing HAP1, H1-ΔV33, and H1-ΔV33-fV33A cells with furin inhibitor (FI) or mock treatment, cells were inoculated with MHV-EFLM or mutant virus MHV-S2′FCS in presence or absence of FI. At 7 hpi the cells were lysed and viral-replication dependent luciferase expression levels were determined. In agreement with previous results

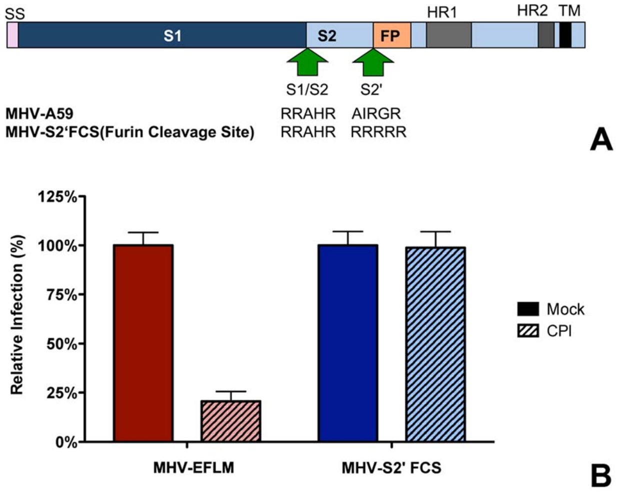

Figure 7. Introduction of a furin cleavage site just upstream of the fusion peptide renders MHV independent of lysosomal proteases. A) Schematic representation of the MHV spike protein. The MHV S proteins are partially processed by furin at the S1/S2 boundary (S1/S2) as indicated by the arrow. The furin cleavage site sequence at this position (RRAHR) is shown. The signal sequence (SS) at the amino-terminal end of the S1 subunit and the approximate positions of the fusion peptide (FP), heptad repeat regions 1 and 2 (HR1 and HR2) and the transmembrane domain (TM) in the S2 subunit are indicated. MHV-S2′FCS virus contains an optimal furin cleavage site (RRRRR) immediately upstream of the FP (S2′, indicated by the arrow. **B**) Effect of pan-lysosomal protease inhibitor (CPI) on MHV and MHV-S2′FCS infection. HeLa-mCC1a cells were pretreated with CPI for 30 min and inoculated at MOI = 0.2 with luciferase expression cassette containing MHV-EFLM or MHV-S2′FCS in the presence of CPI, after which incubations were continued in the presence of CPI until 7 hpi. Infection levels were determined by measuring the luciferase activity in cell lysates relative to mock-treated cells. Error bars represent SEM, n = 3*3.
doi:10.1371/journal.ppat.1004502.g007

(Fig. 5), infection with MHV carrying a wild type S was severely reduced in cells lacking a functional HOPS complex and addition of the FI did not alter this effect (Figure 8, red bars). In contrast, infection with MHV-S2′FCS was not decreased by the lack of a functional HOPS complex. However, FI treatment had a clearly negative effect on this virus, which was much more dramatic in the absence of a functional HOPS complex in H1-ΔV33 cells (Figure 8, blue). In conclusion, MHV-S2′FCS lost the requirement for a functional HOPS complex in parallel with this virus becoming insensitive to the pan-lysosomal protease inhibitor CPI. In contrast to the virus with the wild type S, the mutant virus became sensitive to inhibition of furin cleavage.

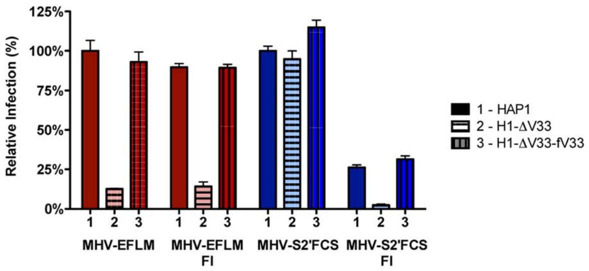

Figure 8. Furin inhibitor reduces infection with MHV-S2′FCS and renders the virus sensitive to endosomal maturation. Haploid HAP1 cells (HAP1), haploid cells lacking VPS33A (H1-ΔV33) or VPS33A-lacking haploid cells retransfected with FLAG-tagged VLP33A (H1-ΔV33-fV33) were infected (MOI = 0.2) with MHV-EFLM (MHV-wt) or MHV-S2′FCS for 7 h. Where indicated, cells were treated with furin inhibitor (FI). Infection levels were determined by measuring the luciferase activity in cell lysates relative to mock-treated cells. Error bars represent SEM, n = 3*3.
doi:10.1371/journal.ppat.1004502.g008

MHV-S2′FCS fuses in early endosomes

To explore MHV-S2′FCS entry requirements further we assessed the effect of RNAi-mediated downregulation of early and late endosome and HOPS complex associated genes. Therefore, HeLa-mCC1a-ΔM15 cells were transfected with each of three different siRNAs per gene for 72 h, after which they were infected with wild type (MHV-EFLM) or mutant (MHV-S2′FCS) S protein containing MHV. At 7 hpi the cells were lysed and viral-replication dependent luciferase expression levels were determined. As found previously (Fig. 1), infection with wild type S protein carrying MHV was reduced after gene silencing of RAB5, RAB7, VPS11, and VPS41 (Figure 9, red bars). On the other hand, infection with MHV-S2′FCS was significantly diminished by downregulation of the early endosomal proteins RAB5B and RAB5C, but not of the late endosomal proteins RAB7A and RAB7B or the HOPS complex components VPS11 and VPS41 (Figure 9, blue bars). Consistently, infections with MHV carrying wild type or mutant S protein were equally blocked by inhibitors of clathrin-mediated endocytosis whereas the virus with the mutant S (MHV-S2′FCS) was much less sensitive to inhibitors of endosomal maturation, including BafA1, or to perturbants of the actin cytoskeleton (Figure S10 in Text S1). From these results we conclude that introduction of a FCS immediately upstream of the FP abolishes the requirement for trafficking of virions to lysosomes and for processing by lysosomal proteases. The resulting virus, which still depends on clathrin-mediated endocytosis, now requires furin cleavage for efficient entry, the enzymes for which occur earlier in the endocytic pathway [78].

Entry of other CoVs

Our results indicate that the protease cleavage site upstream of the spike protein FP is an important determinant of the intracellular site of fusion. To gain more insight into the putative protease cleavage sites in the corresponding region of the S proteins of other CoVs, we analyzed the sequence of this region in several alpha, beta and gamma coronaviruses by performing

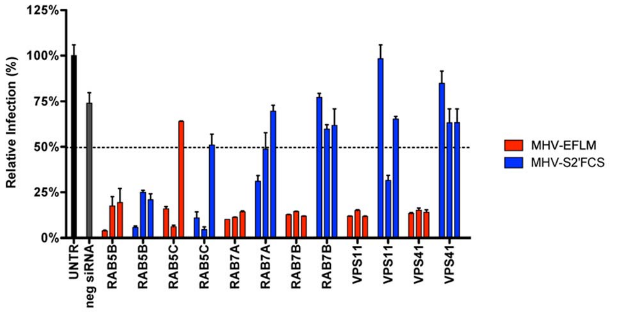

Figure 9. MHV-S2′FCS fuses in early endosomes. siRNA-mediated gene silencing was performed as described in the legend to Figure 1. At 72 h post transfection, HeLa-mCC1a were inoculated with MHV-EFLM or MHV-S2′FCS at MOI = 0.2 and incubated until 7 hpi. Infection levels were determined by measuring the luciferase activity in cell lysates relative to mock-treated cells. Dotted line shows the lower 95% confidence interval of the negative siRNA controls. Error bars represent SEM, n = 3*3.
doi:10.1371/journal.ppat.1004502.g009

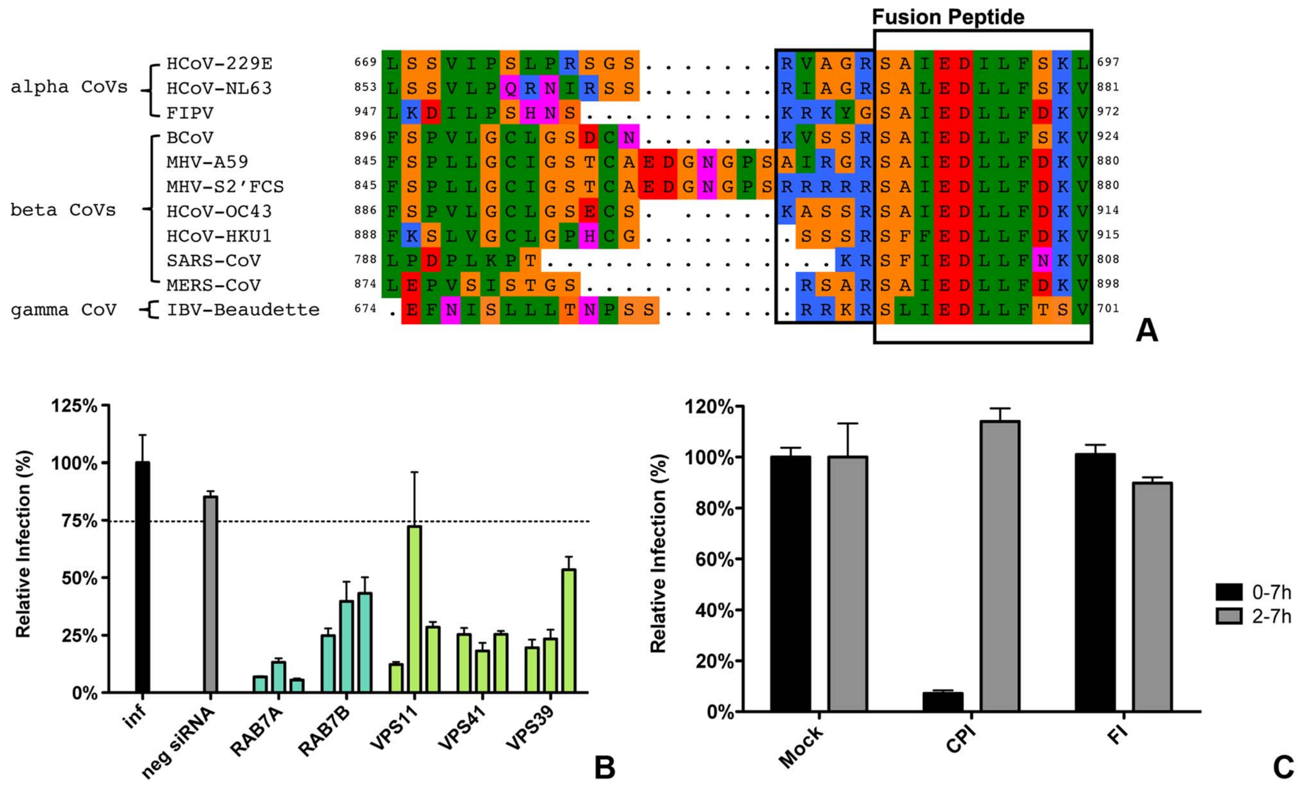

Figure 10. Entry of FIPV. A) Clustal W alignment of spike proteins from several coronaviruses. Displayed is the fusion peptide (boxed) and the area upstream thereof. The area immediately upstream of the fusion peptide that contains the optimal FCS site (RRRRR) in MHV-S2′FCS is also boxed. **B**) siRNA-mediated gene silencing was performed as described in the legend to Figure 1. At 72 h post transfection, HeLa-fAPN cells were inoculated at MOI = 0.2 with luciferase expressing FIPV-RLuc. At 7 hpi infection was determined by measuring the luciferase activity in cell lysates and displayed relative to mock treated infection (inf). Error bars represent SEM, n = 3*3. Dotted line shows the lower 95% confidence interval of the negative siRNA controls. **C**) HeLa-fAPN cells inoculated with FIPV-Rluc at MOI = 0.1 were treated with pan-lysosomal protease inhibitor (CPI) or furin inhibitor (FI) from 30 min prior to 7 h post inoculation (0–7 h) or from 2–7 h post inoculation (2–7 h; hatched bars). Infection levels were determined by measuring the luciferase activity in cell lysates relative to mock-treated cells. Error bars represent SEM, n = 3*3.
doi:10.1371/journal.ppat.1004502.g010

ClustalW sequence alignment. The fusion peptide sequence was found to be highly conserved amongst the different coronaviruses. Also an Arginine residue immediately upstream of the predicted fusion peptide is highly conserved with the exception of FIPV (serotype II). Interestingly, MERS-CoV and IBV-Beaudette contain a minimal furin cleavage site Arg-X-X-Arg just upstream of the fusion peptide (Figure 10A). In analogy with the results obtained with FCS-mutant MHV, we predicted that FIPV and MERS-CoV would differ in their protease inhibitor sensitivity and lysosomal trafficking requirements. To corroborate these findings, we decided to analyze the entry of these two other coronaviruses.

To this end, HeLa cells expressing the FIPV receptor (HeLa-fAPN cells) were subjected to siRNA-mediated downregulation of late endosomal proteins RAB7A and RAB7B or of HOPS complex subunits VPS11, VPS41, and VPS39, followed by inoculation with luciferase expressing FIPV (FIPV-Δ3abcRL; [79]). Infection with FIPV was significantly affected by siRNA-mediated downregulation of proteins required for late endosome-to-lysosome fusion (Figure 10B). Since the requirement for a functional HOPS complex is indicative of fusion in lysosomes, as we observed for MHV, we analyzed whether FIPV requires processing by lysosomal proteases for efficient entry as well. The results indicate that this is indeed the case as FIPV-driven luciferase expression was diminished in the presence of the pan-lysosomal protease inhibitor CPI (Fig. 10C). On the other hand, infection with FIPV was not affected by FI.

As MERS-CoV carries a FCS in its S protein immediately upstream of the FP, we hypothesized this virus not to require trafficking to lysosomes and processing by lysosomal proteases for efficient entry. To test this prediction, Huh-7 cells were pretreated with FI or the pan-lysosomal protease inhibitor CPI for 30 min. Cells were subsequently inoculated with MERS-CoV at a MOI of 0.1 in the presence of these inhibitors. At 8 hpi the cells were fixed and the number of infected cells determined using immunocytochemistry and wide-field microscopy. The results indicate that, in contrast to wild type MHV and FIPV, but similarly to recombinant MHV carrying a FCS immediately upstream of the FP, infection with MERS-CoV is strongly inhibited by the FI but not by CPI (Figure 11), indicating that MERS-CoV does not require trafficking to lysosomes for efficient entry. Based on these results we conclude that the cleavage site in the CoV S protein immediately upstream of the FP is a key determinant of the intracellular site of fusion.

Discussion

The results of this study provide an explanation for several, apparently conflicting results from earlier studies with respect to the process of MHV cell entry, particularly also regarding the necessity of proteolytic cleavage of the CoV S protein. By using a replication-independent fusion assay, we confirmed that MHV entry requires clathrin-mediated endocytosis despite the well-

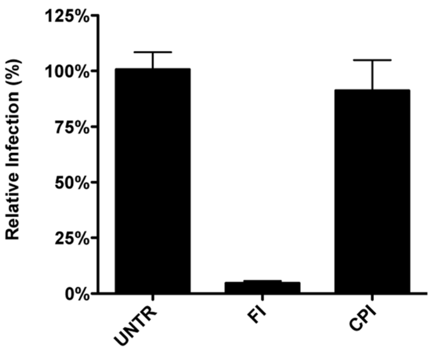

Figure 11. MERS-CoV requires cleavage by furin but not by lysosomal proteases for infection. Huh-7 cells inoculated with MERS-CoV were treated with furin inhibitor (FI) or pan-lysosomal protease inhibitor (CPI) starting from 30 min prior to inoculation. Numbers of infected cells was determined by immunocytochemical staining. Error bars represent SEM, n = 3.
doi:10.1371/journal.ppat.1004502.g011

known ability of the MHV S protein to cause cell-cell fusion at neutral pH. We demonstrate that MHV particles traffic to and fuse in lysosomes. Yet, MHV is much less sensitive to perturbation of the low pH in the endo-/lysosomal system than low pH-dependent control viruses VSV and IAV. Our results additionally indicate that, for fusion to occur, the S protein of MHV requires proteolytic cleavage immediately upstream of the FP, like other class I fusion proteins. Efficient inhibition of MHV entry was only observed using a pan-lysosomal protease inhibitor, and could not be achieved using more specific protease inhibitors. Introduction of an optimal furin cleavage site in the S protein immediately upstream of the FP abolished the requirement for trafficking of virions to lysosomes for fusion. However, this virus still required clathrin-mediated uptake for efficient entry. Consistent with a common mechanism for the entry of CoVs, FIPV, but not MERS-CoV, the latter of which contains a furin cleavage site immediately upstream of the FP, was shown to require trafficking to lysosomes and processing by lysosomal proteases for efficient entry. Based on these results we propose a model in which the cleavage site immediately upstream of the FP is an essential determinant of the intracellular site of CoV fusion (Figure 12).

The importance of clathrin-mediated endocytosis and endosomal trafficking in the entry of MHV was revealed by several complementary approaches. One of these was siRNA-mediated gene silencing. Although - with the exception of RAB7A - knockdown was not monitored at the protein level, we believe this approach firmly demonstrates the importance of novel host factors for several reasons. Validated siRNAs were used and the experimental conditions were confirmed by analyzing the mRNA expression levels of several genes by quantitative RT-PCR. Furthermore, we made use of three independent siRNAs per target gene, and a target was only classified as a hit when at least two out three siRNAs showed the same phenotype. Importantly, our findings were strengthened by targeting multiple proteins per host cell pathway/complex, each time with very similar results. Moreover, hits obtained with the replication-dependent reporter assays were confirmed with our novel replication-independent enzyme complementation entry assay. Also the use of recombinant viruses differing only in their spike proteins enabled us to show that inhibition of virus infection upon siRNA transfection resulted from differences in virus entry and not virus replication. Finally, the results obtained were corroborated by using a large panel of inhibitors and by making use of haploid knockout cells, in which late endosome-to-lysosome trafficking was inhibited.

Our results demonstrate that MHV requires endocytic uptake for virus entry despite the S protein's ability to induce cell-cell fusion at neutral pH. Endocytic uptake is also required for a mutant virus carrying a S protein with a FCS immediately upstream of its FP, despite the relative insensitivity to high concentrations of BafA1. Therefore, the ability of a virus to infect cells in the presence of BafA1 does not necessarily imply virus entry to occur at the cell surface. Also a recombinant MHV carrying the spike protein of MHV-4 (MHV-JHM) was found to enter via clathrin-mediated endocytosis (MHV-S4; Figure S10 in Text S1) despite its ability to cause extensive cell-cell fusion [80–82]. The ability of MHV to cause cell-cell fusion at neutral pH while requiring endocytic uptake for virus-cell fusion suggests different requirements and triggers for these two fusion processes. Similarly, RSV was recently shown to enter cells after endocytic uptake despite the ability of this virus to cause cell-cell fusion [13].

The present study confirms and extends previous publications on MHV entry via clathrin-mediated endocytosis [26,83]. Both siRNAs downregulating clathrin-mediated endocytosis-associated proteins, such as clathrin heavy chain (CLTC) and Dynamin 2 (DNM2), and agents affecting this uptake pathway (Chlopro, Dynasore, Dyngo-4a) were capable of inhibiting infection with MHV. Importantly, these findings could be confirmed in our novel replication-independent virus-cell fusion assay, thereby directly showing an involvement of clathrin-mediated endocytosis in entry of MHV. Analysis of several accessory factors of clathrin-mediated endocytosis showed that clathrin-mediated entry of MHV strain A59 depends on clathrin-adaptor DAB2, but not on EPS15 or AAK1. Previously, clathrin-mediated entry of MHV strain 2 was also shown to be independent of EPS15 [83]. Based on the use of inhibitors, it was earlier concluded that MHV entry depends on cholesterol and lipid-rafts, which may be indicative of caveolae-mediated endocytosis [84,85]. Although our replication-dependent assays indicate a requirement for caveolin 2 (CAV2) for infection, this protein was shown not to be involved in virus entry using our fusion assay. Also depletion of other proteins involved in caveolae-mediated endocytosis, including caveolin 1 (CAV1) and flotillins 1 and 2 (FLOT1 and FLOT2) did not affect MHV infection or fusion. Interestingly, fusion of MHV was severely inhibited by EIPA, an inhibitor of the Na^+/H^+ exchanger NHE1, which is regarded as a hallmark inhibitor of macropinocytosis. Apparently, inhibition of virus entry by EIPA does not prove by itself that a virus enters via this particular pathway. EIPA has been reported to affect several other cellular processes, including actin remodeling, internalization of lipid rafts, distribution of endosomes, and even clathrin-mediated endocytosis [86–90]. Similar to the results obtained with the HeLa cells, also infection of murine LR7 cells was inhibited by compounds interfering with clathrin-mediated endocytosis (Figure S11A in Text S1).

MHV virions require trafficking through the endocytic pathway to lysosomes for efficient entry. Upon clathrin-mediated uptake these virions are temporarily associated with early endosomes as demonstrated by co-localization with RAB5 during live cell imaging. Furthermore, the importance of early endosomes for entry was indicated by siRNA-mediated downregulation of various proteins associated with early endosomes (EEA1, RAB5A, RAB5B, and RAB5C), which inhibited MHV infection, as well as virus-cell fusion. However, only very few MHV particles appeared to fuse in the early endosomes. Live cell imaging indicated fusion largely to occur in late endosomes and/or

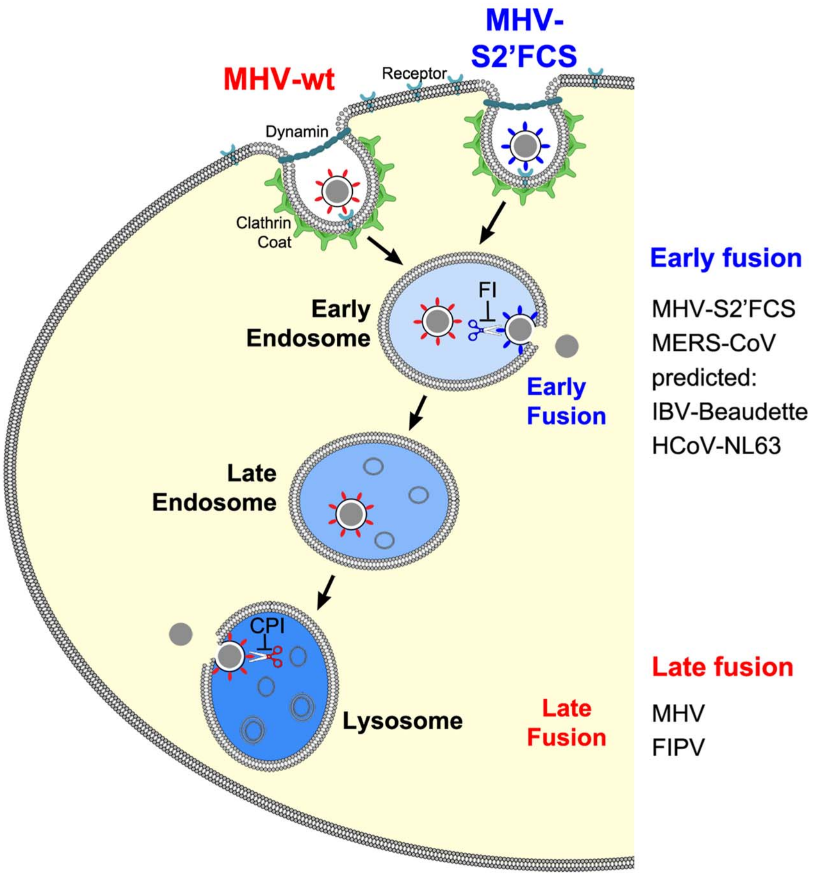

Figure 12. Model of early and late coronavirus fusion. MHV and MHV-S2′FCS are taken up by DAB2-dependent clathrin-mediated endocytosis to end up in RAB5-containing early endosomes. The FCS of MHV-S2′FCS is cleaved by furin or furin-like enzymes to allow fusion of the virus in early endosomes. Trafficking of MHV from late endosomes to lysosomes (RAB7/LAMP1-positive compartments) is required for processing of MHV by lysosomal proteases and viral fusion to occur. We propose that the sequence immediately upstream of the FP is a key determinant of the intracellular site of fusion. MERS-CoV and FIPV enter cells via fusion in early endosomes or lysosomes, respectively. MERS-CoV, which contains a minimal FCS, is inhibited by furin inhibitor (FI) but not by the pan-lysosomal protease inhibitor (CPI). The opposite holds true for FIPV. Based on this model, we predict that IBV strain Beaudette and HCoV-NL63, which contain FCSs (Fig. 10), to fuse in early endosomes in a furin-dependent manner. Other CoVs that do not contain a FCS at this position are predicted to fuse in lysosomes.
doi:10.1371/journal.ppat.1004502.g012

lysosomes. Consistently, depletion of host proteins associated with late endosome and late endosome-to-lysosome maturation (RAB7A, RAB7B, and the HOPS complex subunits VPS11, VPS33A, VPS39 and VPS41) or addition of U18666A, which blocks late endosome-to-lysosome trafficking, were shown to inhibit both infection and virus-cell fusion. The importance of lysosomes for entry was confirmed by using knockout cells lacking a functional HOPS complex (For a review on the HOPS complex see [54]). Interestingly, in these cells lysosomes are clustered in a perinuclear region of the cell rather than dispersed throughout the cytoplasm. Complementation of the missing HOPS subunit restored the normal lysosome distribution and entry of MHV (Figure S7 in Text S1). The importance of late endosome-to-lysosome trafficking for efficient entry was also observed in murine cells (Figure S11C in Text S1) and for MHV-S4 carrying the S protein of MHV-4 (JHM; Figures S10 and S12 in Text S1).

Corroborating the importance of trafficking of MHV virions through the endocytic pathway, perturbation of endosome maturation by the addition of inhibitory agents, such as ammonium chloride, BafA1, Chloroquine, and Monensin inhibited infection and fusion of MHV. Also the importance of the actin and microtubule cytoskeleton - as demonstrated by the inhibition of MHV entry by downregulation of the Arp2/3 complex factors (ACTR2 and ACTR3), of the microtubule-associated transporter dynein (DYNC1H1 and DYNC2H1), or by addition of actin- or microtubule-affecting drugs - may be explained by the documented involvement of the cytoskeleton in endosome maturation (reviewed in [7]). Indeed, entry of MHV-S2′FCS, which presumably fuses in early endosomes, was much less affected by actin-affecting drugs than that of MHV carrying wild type spike proteins (Figure S10 in Text S1). However, we cannot exclude that actin also plays a role in the clathrin-mediated uptake of MHV particles, as has been observed for VSV and other pathogens that depend on clathrin-mediated endocytosis (reviewed in [91]).

MHV particles require trafficking to the low pH environment of lysosomes to achieve membrane fusion. Nevertheless, MHV was much less sensitive to elevation of pH in the endo-/lysosomal system by the addition of BafA1 than viruses known to fuse in early or late endosomes (VSV and IAV). BafA1, an inhibitor of vacuolar-type H^+-ATPase was effective in blocking MHV entry only at high concentrations, which are known to prevent endosomal maturation in addition to the elevation of the pH [66]. The absence of a functional HOPS complex, which is required for late endosome-to-lysosome maturation, did not affect

infection of cells with VSV or IAV, while entry of MHV was severely reduced. Thus, the low pH trigger that mediates entry of VSV and IAV in the endosomal system of these cells, is not sufficient to induce fusion of MHV. Other environmental cues, present in lysosomes only, are apparently required to activate conformational changes in the S protein leading to fusion. Indeed, inhibition of the three major classes of proteases present in the lysosome by CPI effectively prevented MHV fusion. Infection of murine LR7 cells with MHV was also inhibited by CPI (Figure S11B in Text S1). Strikingly, other inhibitors that affect members of a single protease family had none or only little impact on MHV fusion. These results are in consistence with a functional redundancy of protease family members [47,76] and may explain why previous studies using specific lysosome protease inhibitors [27,92] failed to detect entry inhibition. Also, the inhibition of MHV entry by MG132 may be explained by the known ability of the proteasome inhibitor to negatively affect lysosomal proteases [93–95], although we cannot exclude that MG132 affects entry by its interference with lysosomal trafficking [96].

Our results indicate that cleavage of the S protein immediately upstream of the FP is essential for CoV entry and determines the intracellular site of fusion. Although we did not demonstrate cleavage of MHV S at the FP proximal position directly, a recent study found a cleaved form of the MHV S2 subunit to correspond with the fusion-active form [49]. Furthermore, introduction of an optimal FCS at the FP proximal position abolished the entry inhibition by the pan-lysosomal protease inhibitor whilst introducing a dependency on furin-related enzymes. Consistent with the known presence of active furin in early endosomes (reviewed in [78]) the mutant virus no longer required trafficking to late endosomes/lysosomes for entry to occur. However, in the presence of furin inhibitor, entry of this mutant MHV was much more efficient in wild type cells than in cells lacking a functional HOPS complex (Fig. 8), indicating that under certain circumstances lysosomal proteases may play a role in entry of this virus as well. Trafficking of virions to lysosomes was shown to be also important for entry of FIPV, but not of MERS-CoV, in agreement with the latter virus containing a putative FCS immediately upstream of the FP. Correspondingly, entry of FIPV was inhibited by the pan-lysosomal protease inhibitor CPI but not by furin inhibitor, while the reciprocal held true for MERS-CoV. The importance of S protein cleavage downstream of the S1/S2 boundary and upstream of the FP for infection has so far only been demonstrated for SARS-CoV and IBV [40,43,46–48].

Based on the present study and on the work of others, we conclude that cleavage at the FP proximal position is likely to be a general requirement for CoV entry. With the exception of possibly IBV, cleavage at this position does not appear to occur in the virion-producing cell as it is not observed in released virions, but in the target cell (this study; [40,43,47,48]). This suggests that receptor binding or other environmental cues are necessary to render the cleavage site accessible for proteolysis in the intact virion. Also for several other viruses, including RSV [13] and Ebola virus [16], cleavage of the fusion protein upon endocytosis has been shown to be required for entry. Our results furthermore show that cleavage at a FP-proximal position is an important determinant of the intracellular site of fusion. The question remains, however, why some CoVs evolved to fuse in early endosomal vesicles while others require trafficking to lysosomes. In view of the growing number of proteases that have been shown to cleave CoV spike proteins [97], this question should probably be studied in relation to the proteolytic enzymes available in the CoV target tissues and cells *in vivo*.

Materials and Methods

Cells and viruses

Murine LR7 fibroblast [98] and feline FCWF cells (ATCC) were used to propagate the recombinant MHV and FIPV viruses, respectively. HEK293T, MDCK and Vero cells were used to propagate pseudotyped VSVΔG/Luc-G*, *Renilla* luciferase expressing influenza A pseudovirus, or MERS-CoV, respectively, as described previously [71,73,99]. Cells were maintained as monolayer cultures in Dulbecco's modified Eagle's medium (DMEM, Lonza), supplemented with 10% fetal bovine serum (FBS). HeLa-ATCC cells stably expressing murine CEACAM1a (HeLa-mCC1a) and LR7 cells were used for infection experiments with MHV. HeLa-mCC1a cells stably expressing the deficient β-galactosidase ΔM15 (HeLa-mCC1a-ΔM15) were used in the fusion assay. Stable cell lines were generated using a Moloney murine leukemia (MLV) retroviral vector. MLV was produced in HEK293T cells by triple plasmid transfection of a transfer vector containing the ΔM15 or mCC1a gene as well as a puromycin or neomycin resistance marker gene, respectively, in combination with expression vectors encoding the MLV Gag-Pol, and the VSV spike protein G. Upon MLV transduction, stably transduced cells were selected at 2 μg/ml puromycin and/or 0.5 mg/ml G418 (both Sigma), maintenance at 1 μg/ml puromycin and/or 0.5 mg/ml G418 in DMEM, supplemented with 10% FBS. HAP1 cells and the VPS33A knockout derivative thereof (H1-ΔV33) have been described previously [74]. H1-ΔV33 cells were stably transfected with FLAG-tagged VPS33A (H1-ΔV33-fV33) using MLV transduction as described above using a blasticidin resistance marker gene in the transfer vector. Stably transduced cells were selected and maintained at 5 μg/ml blasticidin. HAP1 cells and its derivatives were also provided with mCC1 as described above to allow infection of these cells with MHV.

Chemicals

The MHV fusion inhibitor HR2 peptide has been described before [100] and was synthesized by GenScript. The peptide was diluted in Tris/HCl 50 mM, pH 7.8, 4 μM EGTA at 1 mM stock solution and used at 10 μM final concentration. Fluorescein-di-β-D-galactopyranoside (FDG) (AnaSpec) was dissolved in DMSO resulting in a 20 mM stock solution. Stocks of 700 mM cycloheximide (CHX, Sigma), 125 μM Bafilomycin A1 (BafA1, Enzo Life Sciences), 140 mM Chloroquine (Chloq, Sigma), 120 mM Dynasore (Dyn, Enzo Life Sciences), 15 mM Dyngo-4a (Dyngo, Abcam), 100 mM Ethylisopropyl amiloride (EIPA, Enzo Life Sciences), 1 mM Nocodazole (Noc, Sigma), 1 mM Latrunculin A (LatA, Enzo Life Sciences), 2 mM Jasplakinolide (Jasp, Sigma), 20 mM Cytochalasin B (CytoB, Sigma), 20 mM Cytochalasin D (CytoD, Sigma), 25 mM MG132 (Sigma), 1 mM Brefeldin A (BrefA, Sigma), and 10 mM Furin Inhibitor I (FI, Calbiochem) were prepared in DMSO and diluted 1:1000 in the experiments, except when indicated otherwise. Stocks of 2 M ammonium chloride (NH_4Cl, Fluka), 5 mM AEBSF, 5 mM Leupeptin, 1 mM Camostat, 1 mg/ml Aprotinin (all obtained from Sigma) were prepared in H_2O and used at 1:100 final concentrations. 10 mM chlorpromazine (Chlopro, Sigma), and 20 mM U18666A (Enzo Life Sciences) were prepared in H_2O and used at 1:1000 final concentrations. Stocks of 6 mM Monensin (Mon, Sigma) and 5 mM Phosphoramidon (Sigma) were prepared in methanol (MeOH) and used at 1:1000 and 1:100 final concentrations, respectively. 25 mg/ml cycloheximide (CHX, Sigma) and 5 mM Pepstatin A (Sigma) were prepared in methanol (EtOH) and used at 1:1000 and 1:100 final concentrations, respectively. Solvents EtOH, MeOH, and DMSO were obtained

from Sigma-Aldrich. A stock of 125 μM CPI in PBS was made [76] and used at 5 μM final concentration.

Plasmids

All plasmids were constructed using conventional cloning techniques. The ΔM15 gene was isolated from a DH5 *E. coli* strain by DNA extraction and PCR. The gene was cloned into a pCAGGS vector for (transient) expression and into a MLV-based pQCXIP transfer vector (Clontech), resulting pQCXIP-ΔM15, for the generation of stable cell lines. The gene encoding the MHV receptor mCC1a [101] was cloned into pQCXIN, resulting in pQCXIN-mCC1a. The RNA transcription vectors used for the generation of recombinant MHV using targeted recombination were generated using pMH54 derivatives [98,102]. pMH54 containing a GFP expression cassette between the E and M gene was generated as described previously for firefly luciferase [59]. The transcription vector used to generate MHV-S2′FCS (pXHERLM-S2′FCS+) was generated by site-directed mutagenesis, thereby changing the sequence encoding AIRGR immediately upstream of the FP into a RRRRR-encoding sequence in vector pXHERLM [59] (GCA′ATC′CGA′GGG′CGT to AGA′CGC′CGA′AGG′CGT). The transcription vector used to generate MHV-S4 expressing firefly luciferase, was generated by introducing the firefly luciferase expression cassette between the E and M genes similarly as described previously [59] in a pMH54-derived transcription vector that contains the gene encoding the S protein of MHV-4 (MHV-JHM) [82]. This latter vector was kindly provided by Susan Weiss.

Generation of recombinant/pseudo viruses

Recombinant MHV-EGFPM virus, containing a GFP expression cassette between the E and the M gene, MHV-S2′FCS, containing a *Renilla* luciferase expression cassette between the E and the M gene and a FCS at the FP-proximal position, and MHV-S4 containing the spike gene of MHV-4 (JHM) and a luciferase expression cassette were generated by targeted RNA recombination as described before [98]. Briefly, donor RNA was generated from linearized pMH54-derived transfer vectors described above, and electroporated into FCWF cells infected with interspecies chimeric fMHV coronavirus (an MHV-A59 derivative, in which the ectodomain of the spike protein has been replaced by that of a feline coronavirus, thereby changing host cell tropism). The electroporated FCWF cells were seeded onto a monolayer of LR7 cells. After 24 h of incubation at 37°C, culture supernatant containing progeny viruses was harvested. Genotypes of the recombinant viruses were confirmed after two rounds of plaque purifications. Passage 3 stocks were used in experiments. Generation of MHV-EFLM and MHV-ERLM, containing a firefly or *Renilla* luciferase expression cassette between the E and the M gene, and MHV-αN, containing a N protein tagged with the α-peptide, has been described before [63,103]. Construction of FIPV expressing *Renilla* luciferase was reported previously [79].Recombinant VSVΔG/FLuc-G* pseudovirus was generated as described before [71]. Construction of IAV-WSN pseudovirus expressing *Renilla* luciferase has also been described previously [73]. Viruses were stored in culture medium, supplemented with 25 mM HEPES or upon sucrose cushion purification in TN buffer (10 mM Tris-Cl, pH 7.4, 10 mM NaCl).

siRNA transfections

30,000 HeLa-mCC1a-(ΔM15) cells were seeded one day prior to transfection in a 24-well dish. Using Oligofectamine (Life Technologies) reagent three independent, non-overlapping siRNAs (pre-designed Silencer Select siRNAs from Ambion) per gene were individually transfected into target cells according to the manufacturer's instructions. Transfection mix for one well contained 2.5 μl of 1 μM siRNA and 0.5 μl Oligofectamine in 50 μl OptiMEM (Gibco). Transfection was done in 250 μl final volume of OptiMEM. 4 hours post transfection 125 μl of DMEM, 30% FBS were added. Cells were infected 72 hours post transfection.

qRT-PCR of siRNA-mediated gene knockdowns

HeLa-mCC1a cells were subjected to siRNA-mediated gene knockdown as described above. At 72 hpi cells were harvested by trypsinization, single-cell suspension counted, and collected by centrifugation. Cellular RNA was extracted using the RNeasy Mini Kit (Qiagen). mRNA levels of genes were analyzed by qRT-PCR using a custom designed pair of specific primers to the gene resulting in about 150 bp products. RNA levels were measured using the GoTaq 1-Step RT-qPCR system (Promega) according to the manufacturers' instructions on a LightCycler 480 (Roche). Expression levels were corrected for cell number and viability as determined by the Wst-1 assay (Roche).

Virus infections

Cells were inoculated with MHV-EGFPM at MOI = 0.5 (15–20% infected cells) in DMEM, 2% FBS, for 2 h at 37°C. The inoculum was replaced by warm DMEM, 10% FBS. At 8 hpi, cells infected with MHV-EGFPM were trypsinized and fixed in 4% formaldehyde solution in PBS. Cells were washed and taken up in FACS buffer (2% FBS, 0.05M EDTA, 0.2% NaN_3 in PBS) and GFP expression was quantified by FACS analysis on a FACS Calibur (Benson Dickson) using FlowJo software. Of each sample at least 10,000 cells were analyzed. HeLa, LR7, or HAP1 cells were inoculated with luciferase expressing (pseudo)viruses (MHV-EFLM, VSVΔG/FLuc-G*, IAV-RLuc, MHV-S2′FCS, or FIPV-RLuc, MHV-EFLM-S4 (JHM)) at MOI = 0.2, unless indicated otherwise, in DMEM or IMDM (HAP1), supplemented with 2% FBS at 37°C. At 2 hpi the inoculum was replaced by warm culture medium containing 10% FBS. Cells were lysed at 7 hpi (MHV, VSV, and FIPV) or 16 hpi (IAV) in passive lysis buffer (Promega). Firefly luciferase expression was assessed using the firefly luciferase assay system from Promega or using a homemade system (50 mM tricine, 100 μM EDTA, 2.5 mM $MgSO_4$, 10 mM DTT, 1.25 mM ATP, 12.5 μM D-Luciferin). *Renilla* luciferase expression was assessed using the *Renilla* luciferase assay system (Promega). Light emission was measured on a Centro LB 960 luminometer. When indicated cells were transfected with siRNAs prior to inoculation as described above. Luciferase expression levels (in relative light units, RLU) were corrected for cell number and viability as determined by the Wst-1 assay (Roche). When indicated cells were treated with pharmacological inhibitors starting at 30 min prior to or 2 h post inoculation. Huh-7 cells were inoculated with MERS-CoV at a MOI of 0.1 in FBS-containing DMEM. 8 h post infection, cells were fixed in 4% formaldehyde in PBS. Cells were stained using rabbit anti-SARS-CoV nsp4 antibodies that are cross-reactive for MERS-CoV, according to a standard protocol using a FITC-conjugated swine-anti-rabbit antibody. Number of infected cells was determined by cell counts on a wide-field fluorescent microscope.

Fusion assay using β-galactosidase complementation

The β-galactosidase complementation fusion assay was performed as described previously [63]. Briefly, cells were preloaded with FDG substrate by incubation of adherent target cells with 2.5% FBS, 100 mM FDG, 50% PBS at room temperature. After 3 min incubation an excess of 5% FBS in PBS was added, supernatant removed and replaced by growth medium. After a recovery period of 30 min at 37°C, cells were (mock) treated with

the different inhibitors for 30 min. MHV-αN virus was bound to cells in DMEM with 2%FCS (in the absence or presence of inhibitors) at a MOI = 20 for 90 min at 4°C to synchronize infection, after which cells were shifted to 37°C for 2 h. Cells were trypsinized and transferred to Eppendorf tubes, washed and immediately analyzed by FACS. For experiments with protease inhibitors the cells were loaded with FDG by hypotonic shock after trypsination and collection of the cells. In this case, FDG loaded cells were incubated on ice for 14 h before being analyzed by FACS.

Fluorescent labeling of MHV

MHV wt virus was grown on LR7 cells and purified over a 20% sucrose cushion in TN buffer by centrifugation at 110,000 rcf for 2.5 h. Supernatant was removed and pellet resuspended in 200 µl TN buffer overnight on ice. Concentrated virus solution was subjected to further purification on a Pfefferkorn gradient (10–20%, 25–50%, 50% cushion). After spinning for 1 h at 150,000 rcf a clear virus band was visible. The virus band was collected and diluted in TN buffer. The virus was pelleted by centrifugation at 110,000 rcf for 1 h and resuspended in 200 µl 0.1M sodium phosphate, 0.15M NaCl buffer pH 7.2 overnight on ice. The purified virus solution was labeled using DyLight NHS 488 (Thermo Scientific) according to the manufacturer's instructions. Infectivity of the labeled virus was confirmed by TCID50 analysis and qRT-PCR.

Live-cell microscopy

HeLa-mCC1a cells were seeded into 8-well glass-bottom chambers to reach 60% confluency the next day. Plasmids encoding mRFP-tagged RAB5A or RAB7A, or dsRed-LAMP1 [104] were transfected into the cells one day after seeding using Lipofectamine 2000 (Life Technologies) according to the manufacturer's instructions. 24 h after transfection MHV-DyLight488 was bound to cells on ice at MOI = 20 for 1.5 h in DMEM, 2% FBS. The inoculum was removed and cells washed with cold PBS to remove unbound virus. Warm imaging medium (DMEM without phenol red, 10% FCS) containing 0.008% trypan blue (Invitrogen) was added to the cell chambers. The cell membrane impermeable trypan blue shifts the expression spectrum of cell surface bound particles rendering them undetectable in the 505–530 nm channel (described in [65]). Different low to medium RFP expressing cells were imaged live at 37°C, 5% CO_2 in 10 min time frames from 10 min post warming up to 70 min in 30 s intervals thereby acquiring z-stack images. Each slice was 0.30 µm in thickness, averaging 12–14 slices per stack. For recording a Zeiss Axio Observer Z1 inverse spinning-disk confocal microscope, equipped with full box stage incubation, including CO_2 (Pecon), argon-krypton and helium-neon laser, two Photometrics Evolve 512 back-illuminated electron-multiplying charge-coupled-device (EM-CCD) cameras, and 100×1.46NA Oil alpha Plan Apochromat objective was used. Fluorescence images were exported as.czi files (Zeiss) and subsequently imported into Fiji (ImageJ, NIH).

Upon import into Fiji, color channels were split and saved as 8-bit tagged image file format. Virus movements were manually tracked in x/y or z direction in the green channel using the MTrackJ plugin. Tracks were saved and subsequently loaded onto the red channel. For each virus spot the area underlying a circle of 0.213 μm^2 was measured for its gray mean value. Viruses were considered colocalizing if the gray mean value reached 50% of the maximum. Subsequently red and blue color channels were merged, tracks imported and viruses classified using the viral track. If the virus co-localized with the endosomal vesicle over at least four sequential 30 s frames the virus was categorized as associating. Viruses that, after initial co-localization, separated from the vesicle were classified as 'associating/dissociating'. If a virus particle faded and disappeared (and could not be found in other z-stacks) whilst co-localizing in previous intervals with an endosomal vesicle it was categorized as 'fusing' (Figure S2 and S3 in Text S1). When a viral particle co-localized with endosomal compartments but did neither dissociate nor fade during the 10 min acquisition period it was classified as 'non-fusing'. With this quantification method we analyzed 12 cells for RAB5 with 75 virions in total, 12 cells for RAB7 with 105 virions in total, and 16 cells for LAMP1 with 115 virions in total, acquired over three independent experiments.

Sequence alignment

The sequences of MHV-A59 and MHV-S2′FCS were based on pMH54 sequencing results. Sequences for BCoV (GI: 18033975), FIPV (GI: 556925469), HCoV-OC43 (GI: 530802591), HCoV-HKU1 (GI: 306569687), SARS-CoV (GI: 89474484), MERS-CoV (GI: 510937295), HCoV-229E (GI: 82780499), HCoV-NL63 (GI: 530802144), IBV-Beaudette (GI: 138186) were obtained from NCBI. Alignments were performed over the entire length of the spike proteins using MegAlign (Lasergene DNASTAR) using a ClustalW alignment, gap penalty 10, gap length penalty 0.2, delay divergent sequences 30%, DNA translation weight 0.5, protein weight matrix: PAM series, DNA weight matrix: ClustalW.

Confirmation of siRNA-mediated knockdown of RAB7A

HeLa cells were co-transfected with mRFP-tagged RAB7A similarly as described previously [60]. Briefly, 7'500 HeLa cells were seeded one day prior to transfection in a 96-well plate. Using Oligofectamine (Life Technologies) reagent three independent, non-overlapping RAB7A siRNAs (pre-designed Silencer Select siRNAs from Ambion) per gene were individually transfected into target cells with the mRFP-RAB7A plasmid. Transfection mix for one well contained 2.5 µl of 1 µM siRNA, 10 ng plasmid, and 0.5 µl Oligofectamine in 12.5 µl OptiMEM (Gibco). Transfection was done in 62.5 µl final volume of OptiMEM. 4 hours post transfection 125 µl of DMEM, 30% FBS were added. RFP expression was analyzed 24 h post transfection using an EVOS Cell Imaging System.

Immunostaining of HAP1 cells

Confluent HAP1, H1-ΔV33, and H1-ΔV33-fV33 cells and their stably mCeacam1a expressing counterparts were detached using a cell scraper, homogenized, and fixed. After 30 min incubation in blocking buffer (3% BSA (Sigma), in PBS) for 1 h cells were incubated in 1:100 N-CEACAM-Fc [80] antibody, washed, and stained with 2ry AF488 goat-anti-rabbit antibody (Life Technologies). After washing cells were analyzed by FACS at 10,000 gated single cells per sample.

Western blotting

HAP1 cells were trypsinized and collected by centrifugation at 350 rcf for 10 min. The pellet was resuspended in Laemmli sample buffer containing 100 mM DTT, boiled for 5 min at 95°C and subjected to electrophoresis in 10% acrylamide (Bio-Rad) gels. Viruses were purified and concentrated over a 20% sucrose cushion (in TN buffer) at 110,000 rcf. Pelleted virus was resuspended in TN buffer overnight on ice. After addition of Laemmli sample buffer (1× final concentration, 100 mM DTT), samples were boiled for 5 min at 95°C and subjected to electrophoresis in 7% acrylamide (Bio-Rad) gels. Upon transfer to a nitrocellulose membrane (Millipore), the presence of cellular and viral proteins was probed

with antibodies against GM130 (rabbit pAb, Abcam), FLAG (HRP-labeled mouse anti-FLAG mAb, Sigma) or the S2 subunit of MHV A59 [105] (mouse anti-S2 mAb) diluted 1:1000. When necessary, the blots were subsequently incubated with HRP-labeled rabbit anti-mouse or swine anti-rabbit antibodies (both diluted 1:5000; DAKO). Binding of HRP-labeled antibodies was visualized using Amersham ECL Western blotting substrate (GE Healthcare Life Sciences) according to the manufacturer's instructions.

Immunofluorescence analysis of HAP1 cells

To image the localization of LAMP1 in HAP1, H1-ΔV33, and H1-ΔV33A-fV33, cells the cells were seeded onto coverslips one day prior to staining. Cells were fixed in 4% formaldehyde in PBS for 15 min at RT, washed with PBS, and subsequently permeabilized in PBS containing 0.1% Triton-X-100 for 10 min. Cells were incubated with antibody against LAMP1 (rabbit anti-LAMP1 pAb, 1:100 dilution; Abcam) in 3% BSA in PBS followed by incubation with secondary antibodies coupled to AF488, AF-568 phalloidin, and DAPI (all Life Technologies). The samples were analyzed using a confocal laser-scanning microscope (Leica SPE-II).

Growth curves of recombinant viruses

LR7 cells were infected at MOI = 0.1 or MOI = 4.0 with MHV-ERLM or MHV-S2′FCS in DMEM containing 2% FBS and 25 mM HEPES (infection medium). After 3 h of infection supernatant was replaced by fresh infection medium and infection was allowed to progress over a period of 24 h. Every 3 h a small sample of the culture supernatant was collected and immediately frozen. The samples were subsequently analyzed in TCID50 assays on LR7 cells and subjected to qRT-PCR analysis to quantify virion production. Therefore viral RNA was extracted from the samples using the QIAamp Viral RNA Mini Kit (Qiagen). The relative amount of viral RNA present was determined with a LightCycler 480 using LightCycler 480 RNA Master Hydrolysis kit (Roche Applied Biosciences) and specific primers and probe targeted against the MHV 1b gene by comparison with a standard curve.

Gene identification numbers

Gene	SwissProt ID
AAK1	Q2M2I8
ACTR2	P61160
ACTR3	P61158
CAV1	Q03135
CAV2	P51636
CLTC	Q00610
DAB2	P98082
DNM1	Q05193
DNM2	P50570
DYNC1H1	Q14204
DYNC2H1	Q8NCM8
EPS15	P42566
FLOT1	O75955
FLOT2	Q14254
LAMP1	P11279
MYO6	Q9UM54
NSF	P46459
PAK1	Q13153
RAB5A	P20339
RAB5B	P61020
RAB5C	P51148
RAB7A	P51149
RAB7B	Q96AH8
SNX1	Q13596
VCL	P18206
VPS11	Q9H270
VPS33A	Q96AX1
VPS39	Q96JC1
VPS41	P49754

Supporting Information

Text S1 Supporting information.
(PDF)

Acknowledgments

We would like to thank Dr. Thijn Brummelkamp, Biochemistry Division, Netherlands Cancer Institute, Amsterdam, The Netherlands, for providing us with HAP1 and HAP1-ΔVPS33A cells, as well as pMX-FLAG-VPS33A plasmid, Dr. Susan Weiss, University of Pennsylvania, Philadelphia PA, USA, for providing us with the pMH54 derived transcription vector that contains the spike gene of MHV-4, and Dr. Tom Gallagher, Department for Microbiology and Immunobiology, Stritch School of Medicine, Lyola Univeristy, Chicago IL, USA for providing us with N-CEACAM-Fc antibody. We also would like to thank Prof. Dr. Ari Helenius and the Light Microscopy and Screening Center, Institute of Biochemistry, Swiss Federal Institute of Technology Zurich, Zurich, Switzerland, for providing us with the possibility to carry out light microscopy experiments in their facilities and providing us with scientific input.

Author Contributions

Conceived and designed the experiments: CB MHV BLH LP PJMR BJB CAMdH. Performed the experiments: CB MHV BLH. Analyzed the data: CB MHV. Contributed reagents/materials/analysis tools: OW SIvK LP BLH. Wrote the paper: CB PJMR BJB CAMdH. Provided scientific input: OW FJvK LP PJMR BJB CAMdH.

References

1. Fuller AO, Spear PG (1987) Anti-glycoprotein D antibodies that permit adsorption but block infection by herpes simplex virus 1 prevent virion-cell fusion at the cell surface. Proceedings of the National Academy of Sciences of the United States of America 84: 5454–5458.
2. Sodeik B, Ebersold MW, Helenius A (1997) Microtubule-mediated transport of incoming herpes simplex virus 1 capsids to the nucleus. The Journal of cell biology 136: 1007–1021.
3. Okada Y (1969) Factors in fusion of cells by HVJ. Current topics in microbiology and immunology 48: 102–128.
4. Permanyer M, Ballana E, Este JA (2010) Endocytosis of HIV: anything goes. Trends in microbiology 18: 543–551.
5. Stein BS, Gowda SD, Lifson JD, Penhallow RC, Bensch KG, et al. (1987) pH-independent HIV entry into CD4-positive T cells via virus envelope fusion to the plasma membrane. Cell 49: 659–668.
6. Authier F, Posner BI, Bergeron JJ (1996) Endosomal proteolysis of internalized proteins. FEBS letters 389: 55–60.
7. Huotari J, Helenius A (2011) Endosome maturation. The EMBO journal 30: 3481–3500.
8. Plemper RK (2011) Cell entry of enveloped viruses. Current opinion in virology 1: 92–100.
9. Sieczkarski SB, Whittaker GR (2003) Differential requirements of Rab5 and Rab7 for endocytosis of influenza and other enveloped viruses. Traffic 4: 333–343.
10. Skehel JJ, Bayley PM, Brown EB, Martin SR, Waterfield MD, et al. (1982) Changes in the conformation of influenza virus hemagglutinin at the pH optimum of virus-mediated membrane fusion. Proceedings of the National Academy of Sciences of the United States of America 79: 968–972.
11. Carneiro FA, Ferradosa AS, Da Poian AT (2001) Low pH-induced conformational changes in vesicular stomatitis virus glycoprotein involve dramatic structure reorganization. The Journal of biological chemistry 276: 62–67.
12. White J, Matlin K, Helenius A (1981) Cell fusion by Semliki Forest, influenza, and vesicular stomatitis viruses. The Journal of cell biology 89: 674–679.

13. Krzyzaniak MA, Zumstein MT, Gerez JA, Picotti P, Helenius A (2013) Host cell entry of respiratory syncytial virus involves macropinocytosis followed by proteolytic activation of the F protein. PLoS pathogens 9: e1003309.
14. Wool-Lewis RJ, Bates P (1999) Endoproteolytic processing of the ebola virus envelope glycoprotein: cleavage is not required for function. Journal of virology 73: 1419–1426.
15. Zimmer G, Budz L, Herrler G (2001) Proteolytic activation of respiratory syncytial virus fusion protein. Cleavage at two furin consensus sequences. The Journal of biological chemistry 276: 31642–31650.
16. Chandran K, Sullivan NJ, Felbor U, Whelan SP, Cunningham JM (2005) Endosomal proteolysis of the Ebola virus glycoprotein is necessary for infection. Science 308: 1643–1645.
17. Peiris JS, Lai ST, Poon LL, Guan Y, Yam LY, et al. (2003) Coronavirus as a possible cause of severe acute respiratory syndrome. Lancet 361: 1319–1325.
18. Zaki AM, van Boheemen S, Bestebroer TM, Osterhaus AD, Fouchier RA (2012) Isolation of a novel coronavirus from a man with pneumonia in Saudi Arabia. N Engl J Med 367: 1814–1820.
19. de Haan CA, Rottier PJ (2005) Molecular interactions in the assembly of coronaviruses. Advances in virus research 64: 165–230.
20. Bosch BJ, van der Zee R, de Haan CA, Rottier PJ (2003) The coronavirus spike protein is a class I virus fusion protein: structural and functional characterization of the fusion core complex. Journal of virology 77: 8801–8811.
21. Inoue Y, Tanaka N, Tanaka Y, Inoue S, Morita K, et al. (2007) Clathrin-dependent entry of severe acute respiratory syndrome coronavirus into target cells expressing ACE2 with the cytoplasmic tail deleted. Journal of virology 81: 8722–8729.
22. Wang H, Yang P, Liu K, Guo F, Zhang Y, et al. (2008) SARS coronavirus entry into host cells through a novel clathrin- and caveolae-independent endocytic pathway. Cell research 18: 290–301.
23. Regan AD, Shraybman R, Cohen RD, Whittaker GR (2008) Differential role for low pH and cathepsin-mediated cleavage of the viral spike protein during entry of serotype II feline coronaviruses. Veterinary microbiology 132: 235–248.
24. Van Hamme E, Dewerchin HL, Cornelissen E, Verhasselt B, Nauwynck HJ (2008) Clathrin- and caveolae-independent entry of feline infectious peritonitis virus in monocytes depends on dynamin. The Journal of general virology 89: 2147–2156.
25. Nomura R, Kiyota A, Suzaki E, Kataoka K, Ohe Y, et al. (2004) Human coronavirus 229E binds to CD13 in rafts and enters the cell through caveolae. Journal of virology 78: 8701–8708.
26. Eifart P, Ludwig K, Bottcher C, de Haan CA, Rottier PJ, et al. (2007) Role of endocytosis and low pH in murine hepatitis virus strain A59 cell entry. Journal of virology 81: 10758–10768.
27. Qiu Z, Hingley ST, Simmons G, Yu C, Das Sarma J, et al. (2006) Endosomal proteolysis by cathepsins is necessary for murine coronavirus mouse hepatitis virus type 2 spike-mediated entry. Journal of virology 80: 5768–5776.
28. Stauber R, Pfleiderera M, Siddell S (1993) Proteolytic cleavage of the murine coronavirus surface glycoprotein is not required for fusion activity. The Journal of general virology 74 (Pt 2): 183–191.
29. Sturman LS, Ricard CS, Holmes KV (1985) Proteolytic cleavage of the E2 glycoprotein of murine coronavirus: activation of cell-fusing activity of virions by trypsin and separation of two different 90K cleavage fragments. Journal of virology 56: 904–911.
30. de Haan CA, Stadler K, Godeke GJ, Bosch BJ, Rottier PJ (2004) Cleavage inhibition of the murine coronavirus spike protein by a furin-like enzyme affects cell-cell but not virus-cell fusion. Journal of virology 78: 6048–6054.
31. Frana MF, Behnke JN, Sturman LS, Holmes KV (1985) Proteolytic cleavage of the E2 glycoprotein of murine coronavirus: host-dependent differences in proteolytic cleavage and cell fusion. Journal of virology 56: 912–920.
32. Luytjes W, Sturman LS, Bredenbeek PJ, Charite J, van der Zeijst BA, et al. (1987) Primary structure of the glycoprotein E2 of coronavirus MHV-A59 and identification of the trypsin cleavage site. Virology 161: 479–487.
33. Ricard CS, Sturman LS (1985) Isolation of the subunits of the coronavirus envelope glycoprotein E2 by hydroxyapatite high-performance liquid chromatography. Journal of chromatography 326: 191–197.
34. Gombold JL, Hingley ST, Weiss SR (1993) Fusion-defective mutants of mouse hepatitis virus A59 contain a mutation in the spike protein cleavage signal. Journal of virology 67: 4504–4512.
35. Leparc-Goffart I, Hingley ST, Chua MM, Jiang X, Lavi E, et al. (1997) Altered pathogenesis of a mutant of the murine coronavirus MHV-A59 is associated with a Q159L amino acid substitution in the spike protein. Virology 239: 1–10.
36. Matsuyama S, Taguchi F (2009) Two-step conformational changes in a coronavirus envelope glycoprotein mediated by receptor binding and proteolysis. Journal of virology 83: 11133–11141.
37. Simmons G, Gosalia DN, Rennekamp AJ, Reeves JD, Diamond SL, et al. (2005) Inhibitors of cathepsin L prevent severe acute respiratory syndrome coronavirus entry. Proceedings of the National Academy of Sciences of the United States of America 102: 11876–11881.
38. Bosch BJ, Bartelink W, Rottier PJ (2008) Cathepsin L functionally cleaves the severe acute respiratory syndrome coronavirus class I fusion protein upstream of rather than adjacent to the fusion peptide. Journal of virology 82: 8887–8890.
39. Simmons G, Reeves JD, Rennekamp AJ, Amberg SM, Piefer AJ, et al. (2004) Characterization of severe acute respiratory syndrome-associated coronavirus (SARS-CoV) spike glycoprotein-mediated viral entry. Proceedings of the National Academy of Sciences of the United States of America 101: 4240–4245.
40. Belouzard S, Chu VC, Whittaker GR (2009) Activation of the SARS coronavirus spike protein via sequential proteolytic cleavage at two distinct sites. Proceedings of the National Academy of Sciences of the United States of America 106: 5871–5876.
41. Matsuyama S, Ujike M, Morikawa S, Tashiro M, Taguchi F (2005) Protease-mediated enhancement of severe acute respiratory syndrome coronavirus infection. Proceedings of the National Academy of Sciences of the United States of America 102: 12543–12547.
42. Belouzard S, Madu I, Whittaker GR (2010) Elastase-mediated activation of the severe acute respiratory syndrome coronavirus spike protein at discrete sites within the S2 domain. The Journal of biological chemistry 285: 22758–22763.
43. Kam YW, Okumura Y, Kido H, Ng LF, Bruzzone R, et al. (2009) Cleavage of the SARS coronavirus spike glycoprotein by airway proteases enhances virus entry into human bronchial epithelial cells in vitro. PloS one 4: e7870.
44. Bertram S, Glowacka I, Muller MA, Lavender H, Gnirss K, et al. (2011) Cleavage and activation of the severe acute respiratory syndrome coronavirus spike protein by human airway trypsin-like protease. Journal of virology 85: 13363–13372.
45. Shulla A, Heald-Sargent T, Subramanya G, Zhao J, Perlman S, et al. (2011) A transmembrane serine protease is linked to the severe acute respiratory syndrome coronavirus receptor and activates virus entry. Journal of virology 85: 873–882.
46. Yamada Y, Liu DX (2009) Proteolytic activation of the spike protein at a novel RRRR/S motif is implicated in furin-dependent entry, syncytium formation, and infectivity of coronavirus infectious bronchitis virus in cultured cells. Journal of virology 83: 8744–8758.
47. Matthews SP, Werber I, Deussing J, Peters C, Reinheckel T, et al. (2010) Distinct protease requirements for antigen presentation in vitro and in vivo. Journal of immunology 184: 2423–2431.
48. Watanabe R, Matsuyama S, Shirato K, Maejima M, Fukushi S, et al. (2008) Entry from the cell surface of severe acute respiratory syndrome coronavirus with cleaved S protein as revealed by pseudotype virus bearing cleaved S protein. Journal of virology 82: 11985–11991.
49. Wicht O, Burkard C, de Haan CA, van Kuppeveld FJ, Rottier PJ, et al. (2014) Identification and characterization of a proteolytically primed form of the murine coronavirus spike proteins after fusion with the target cell. Journal of virology 88: 4943–4952.
50. Snijder B, Sacher R, Ramo P, Liberali P, Mench K, et al. (2012) Single-cell analysis of population context advances RNAi screening at multiple levels. Molecular systems biology 8: 579.
51. Gouin E, Welch MD, Cossart P (2005) Actin-based motility of intracellular pathogens. Current opinion in microbiology 8: 35–45.
52. May RC (2001) The Arp2/3 complex: a central regulator of the actin cytoskeleton. Cellular and molecular life sciences: CMLS 58: 1607–1626.
53. Pfeffer SR (2013) Rab GTPase regulation of membrane identity. Current opinion in cell biology 25: 414–419.
54. Balderhaar HJ, Ungermann C (2013) CORVET and HOPS tethering complexes - coordinators of endosome and lysosome fusion. Journal of cell science 126: 1307–1316.
55. Bonifacino JS, Hurley JH (2008) Retromer. Current opinion in cell biology 20: 427–436.
56. DeMali KA, Burridge K (2003) Coupling membrane protrusion and cell adhesion. Journal of cell science 116: 2389–2397.
57. Bagrodia S, Cerione RA (1999) Pak to the future. Trends in cell biology 9: 350–355.
58. Robinson LJ, Aniento F, Gruenberg J (1997) NSF is required for transport from early to late endosomes. Journal of cell science 110 (Pt 17): 2079–2087.
59. de Haan CA, van Genne L, Stoop JN, Volders H, Rottier PJ (2003) Coronaviruses as vectors: position dependence of foreign gene expression. Journal of virology 77: 11312–11323.
60. Verheije MH, Raaben M, Mari M, Te Lintelo EG, Reggiori F, et al. (2008) Mouse hepatitis coronavirus RNA replication depends on GBF1-mediated ARF1 activation. PLoS pathogens 4: e1000088.
61. Raaben M, Posthuma CC, Verheije MH, te Lintelo EG, Kikkert M, et al. (2010) The ubiquitin-proteasome system plays an important role during various stages of the coronavirus infection cycle. Journal of virology 84: 7869–7879.
62. Huynh KK, Gershenzon E, Grinstein S (2008) Cholesterol accumulation by macrophages impairs phagosome maturation. The Journal of biological chemistry 283: 35745–35755.
63. Burkard C, Bloyet LM, Wicht O, van Kuppeveld FJ, Rottier PJ, et al. (2014) Dissecting Virus Entry: Replication-Independent Analysis of Virus Binding, Internalization, and Penetration Using Minimal Complementation of beta-Galactosidase. PloS one 9: e101762.
64. Langley KE, Villarejo MR, Fowler AV, Zamenhof PJ, Zabin I (1975) Molecular basis of beta-galactosidase alpha-complementation. Proceedings of the National Academy of Sciences of the United States of America 72: 1254–1257.
65. Engel S, Heger T, Mancini R, Herzog F, Kartenbeck J, et al. (2011) Role of endosomes in simian virus 40 entry and infection. Journal of virology 85: 4198–4211.

66. Bayer N, Schober D, Prchla E, Murphy RF, Blaas D, et al. (1998) Effect of bafilomycin A1 and nocodazole on endocytic transport in HeLa cells: implications for viral uncoating and infection. Journal of virology 72: 9645–9655.
67. Johannsdottir HK, Mancini R, Kartenbeck J, Amato L, Helenius A (2009) Host cell factors and functions involved in vesicular stomatitis virus entry. Journal of virology 83: 440–453.
68. Le Blanc I, Luyet PP, Pons V, Ferguson C, Emans N, et al. (2005) Endosome-to-cytosol transport of viral nucleocapsids. Nature cell biology 7: 653–664.
69. Matos PM, Marin M, Ahn B, Lam W, Santos NC, et al. (2013) Anionic lipids are required for vesicular stomatitis virus G protein-mediated single particle fusion with supported lipid bilayers. The Journal of biological chemistry 288: 12416–12425.
70. Skehel JJ, Wiley DC (2000) Receptor binding and membrane fusion in virus entry: the influenza hemagglutinin. Annual review of biochemistry 69: 531–569.
71. Tani H, Komoda Y, Matsuo E, Suzuki K, Hamamoto I, et al. (2007) Replication-competent recombinant vesicular stomatitis virus encoding hepatitis C virus envelope proteins. Journal of virology 81: 8601–8612.
72. Whitt MA (2010) Generation of VSV pseudotypes using recombinant DeltaG-VSV for studies on virus entry, identification of entry inhibitors, and immune responses to vaccines. Journal of virological methods 169: 365–374.
73. Konig R, Stertz S, Zhou Y, Inoue A, Hoffmann HH, et al. (2010) Human host factors required for influenza virus replication. Nature 463: 813–817.
74. Carette JE, Raaben M, Wong AC, Herbert AS, Obernosterer G, et al. (2011) Ebola virus entry requires the cholesterol transporter Niemann-Pick C1. Nature 477: 340–343.
75. Tveten K, Ranheim T, Berge KE, Leren TP, Kulseth MA (2009) The effect of bafilomycin A1 and protease inhibitors on the degradation and recycling of a Class 5-mutant LDLR. Acta biochimica et biophysica Sinica 41: 246–255.
76. van Kasteren SI, Berlin I, Colbert JD, Keane D, Ovaa H, et al. (2011) A multifunctional protease inhibitor to regulate endolysosomal function. ACS chemical biology 6: 1198–1204.
77. White JM, Delos SE, Brecher M, Schornberg K (2008) Structures and mechanisms of viral membrane fusion proteins: multiple variations on a common theme. Critical reviews in biochemistry and molecular biology 43: 189–219.
78. Thomas G (2002) Furin at the cutting edge: from protein traffic to embryogenesis and disease. Nature reviews Molecular cell biology 3: 753–766.
79. de Haan CA, Haijema BJ, Boss D, Heuts FW, Rottier PJ (2005) Coronaviruses as vectors: stability of foreign gene expression. Journal of virology 79: 12742–12751.
80. Gallagher TM (1997) A role for naturally occurring variation of the murine coronavirus spike protein in stabilizing association with the cellular receptor. Journal of virology 71: 3129–3137.
81. Krueger DK, Kelly SM, Lewicki DN, Ruffolo R, Gallagher TM (2001) Variations in disparate regions of the murine coronavirus spike protein impact the initiation of membrane fusion. Journal of virology 75: 2792–2802.
82. Phillips JJ, Chua MM, Lavi E, Weiss SR (1999) Pathogenesis of chimeric MHV4/MHV-A59 recombinant viruses: the murine coronavirus spike protein is a major determinant of neurovirulence. Journal of virology 73: 7752–7760.
83. Pu Y, Zhang X (2008) Mouse hepatitis virus type 2 enters cells through a clathrin-mediated endocytic pathway independent of Eps15. Journal of virology 82: 8112–8123.
84. Choi KS, Aizaki H, Lai MM (2005) Murine coronavirus requires lipid rafts for virus entry and cell-cell fusion but not for virus release. Journal of virology 79: 9862–9871.
85. Thorp EB, Gallagher TM (2004) Requirements for CEACAMs and cholesterol during murine coronavirus cell entry. Journal of virology 78: 2682–2692.
86. Fretz M, Jin J, Conibere R, Penning NA, Al-Taei S, et al. (2006) Effects of Na+/H+ exchanger inhibitors on subcellular localisation of endocytic organelles and intracellular dynamics of protein transduction domains HIV-TAT peptide and octaarginine. Journal of controlled release: official journal of the Controlled Release Society 116: 247–254.
87. Lagana A, Vadnais J, Le PU, Nguyen TN, Laprade R, et al. (2000) Regulation of the formation of tumor cell pseudopodia by the Na(+)/H(+) exchanger NHE1. Journal of cell science 113 (Pt 20): 3649–3662.
88. Meier O, Boucke K, Hammer SV, Keller S, Stidwill RP, et al. (2002) Adenovirus triggers macropinocytosis and endosomal leakage together with its clathrin-mediated uptake. The Journal of cell biology 158: 1119–1131.
89. Wadia JS, Stan RV, Dowdy SF (2004) Transducible TAT-HA fusogenic peptide enhances escape of TAT-fusion proteins after lipid raft macropinocytosis. Nature medicine 10: 310–315.
90. Ivanov AI, Nusrat A, Parkos CA (2004) Endocytosis of epithelial apical junctional proteins by a clathrin-mediated pathway into a unique storage compartment. Molecular biology of the cell 15: 176–188.
91. Kaksonen M, Toret CP, Drubin DG (2006) Harnessing actin dynamics for clathrin-mediated endocytosis. Nature reviews Molecular cell biology 7: 404–414.
92. Huang IC, Bosch BJ, Li F, Li W, Lee KH, et al. (2006) SARS coronavirus, but not human coronavirus NL63, utilizes cathepsin L to infect ACE2-expressing cells. The Journal of biological chemistry 281: 3198–3203.
93. Lee DH, Goldberg AL (1998) Proteasome inhibitors: valuable new tools for cell biologists. Trends in cell biology 8: 397–403.
94. Tawa NE, Jr., Odessey R, Goldberg AL (1997) Inhibitors of the proteasome reduce the accelerated proteolysis in atrophying rat skeletal muscles. The Journal of clinical investigation 100: 197–203.
95. van Kerkhof P, Alves dos Santos CM, Sachse M, Klumperman J, Bu G, et al. (2001) Proteasome inhibitors block a late step in lysosomal transport of selected membrane but not soluble proteins. Molecular biology of the cell 12: 2556–2566.
96. Zaarur N, Meriin AB, Bejarano E, Xu X, Gabai VL, et al. (2014) Proteasome failure promotes positioning of lysosomes around the aggresome via local block of microtubule-dependent transport. Molecular and cellular biology 34: 1336–1348.
97. Belouzard S, Millet JK, Licitra BN, Whittaker GR (2012) Mechanisms of coronavirus cell entry mediated by the viral spike protein. Viruses 4: 1011–1033.
98. Kuo L, Godeke GJ, Raamsman MJ, Masters PS, Rottier PJ (2000) Retargeting of coronavirus by substitution of the spike glycoprotein ectodomain: crossing the host cell species barrier. J Virol 74: 1393–1406.
99. van Boheemen S, de Graaf M, Lauber C, Bestebroer TM, Raj VS, et al. (2012) Genomic characterization of a newly discovered coronavirus associated with acute respiratory distress syndrome in humans. mBio 3.
100. Bosch BJ, van der Zee R, de Haan CA, Rottier PJ (2003) The coronavirus spike protein is a class I virus fusion protein: structural and functional characterization of the fusion core complex. J Virol 77: 8801–8811.
101. Dveksler GS, Pensiero MN, Cardellichio CB, Williams RK, Jiang GS, et al. (1991) Cloning of the mouse hepatitis virus (MHV) receptor: expression in human and hamster cell lines confers susceptibility to MHV. Journal of virology 65: 6881–6891.
102. de Haan CA, Haijema BJ, Masters PS, Rottier PJ (2008) Manipulation of the coronavirus genome using targeted RNA recombination with interspecies chimeric coronaviruses. Methods Mol Biol 454: 229–236.
103. Kuo L, Godeke GJ, Raamsman MJ, Masters PS, Rottier PJ (2000) Retargeting of coronavirus by substitution of the spike glycoprotein ectodomain: crossing the host cell species barrier. Journal of virology 74: 1393–1406.
104. Vonderheit A, Helenius A (2005) Rab7 associates with early endosomes to mediate sorting and transport of Semliki forest virus to late endosomes. PLoS biology 3: e233.
105. Schulze H, Kolter T, Sandhoff K (2009) Principles of lysosomal membrane degradation: Cellular topology and biochemistry of lysosomal lipid degradation. Biochimica et biophysica acta 1793: 674–683.
